# The theoretical impact of AI-based quality evaluation of short-video health information on public cognition and treatment adherence: a case study of denosumab combined with PD-1/PD-L1 therapy for lung cancer bone metastasis

**DOI:** 10.3389/fpubh.2025.1724546

**Published:** 2026-01-06

**Authors:** Jia-wen Wang, Jian-Jun Xun, Fei-Fei Zhao

**Affiliations:** Department of Orthopedics, The Fourth Hospital of Hebei Medical University, Shijiazhuang, Hebei, China

**Keywords:** AI evaluation, algorithmic echo chamber, cognitive bias, denosumab, health information quality, immune checkpoint inhibitors, lung cancer bone metastasis, short-video platforms

## Abstract

**Background:**

Bone metastasis occurs in 30–40% of patients with advanced non-small cell lung cancer (NSCLC), and denosumab combined with PD-1/PD-L1 inhibitors has emerged as a promising treatment strategy. However, the “algorithmic echo chamber” effect on short-video platforms may distort patient cognition and treatment decision-making.

**Methods:**

A cross-sectional study was conducted using a custom-developed web crawler to collect 1,369 videos from Bilibili, Douyin, and Xiaohongshu. A total of 402 videos were included after a three-tier keyword filtering process. An AI-based evaluation system built upon the doubao-seed-1.6 model was established, integrating three international standards—Global Quality Score (GQS), Journal of the American Medical Association (JAMA) benchmark criteria, and the modified DISCERN tool—to assess multidimensional information quality. Kruskal–Wallis tests and Spearman correlation analyses were performed to explore inter-platform differences and the relationship between information quality and user engagement metrics.

**Results:**

Overall video quality was substantially below professional medical standards: the mean GQS was 2.84 ± 1.06 (56.8% of the full score), JAMA was 0.34 ± 0.57 (8.5%), and modified DISCERN was 1.55 ± 0.69 (31.0%). Significant quality differences were observed across platforms (*p* < 0.001, Cohen’s d = 0.6–0.8): Douyin ranked highest, followed by Xiaohongshu, with Bilibili lowest. Correlation between user engagement and content quality was extremely weak (R^2^ = 0.004, r = 0.062), indicating substantial decoupling—high engagement did not equate to high-quality content. Medical professionals accounted for only 25.6% of content creators, while patient-generated content reached 52.2%. Evidence-based treatment information comprised merely 20.0–26.7%, whereas misleading or inaccurate claims accounted for 6.7–13.3%.

**Conclusion:**

From a behavioral and cognitive perspective, the low quality of immune-oncology information on short-video platforms, coupled with algorithm-driven amplification of high-engagement but low-quality content, may exacerbate cognitive bias, potentially increasing clinical safety risks such as insufficient hypocalcemia monitoring and inadequate MRONJ prevention. Establishing a professional governance and oversight system is urgently required.

## 1 Introduction

Lung cancer remains the leading cause of cancer-related mortality worldwide, with more than half of patients diagnosed at a metastatic stage and a low 5-year survival rate of only approximately 5% (1). Bone metastasis is particularly common among patients with advanced non-small cell lung cancer (NSCLC), occurring in up to 30–40% of cases (2). Excessive osteoclast activation promotes osteolytic destruction, while growth factors such as TGF-β and IGF released from bone resorption further enhance tumor proliferation. Additionally, bone marrow stromal cells, myeloid-derived suppressor cells (MDSCs), and regulatory T cells (Tregs) contribute to an immunosuppressive tumor microenvironment, facilitating immune escape. Together, these mechanisms form a self-perpetuating “vicious cycle” that worsens clinical outcomes (3–5).

Denosumab, a fully human monoclonal antibody, specifically binds to receptor activator of nuclear factor-κB ligand (RANKL), thereby inhibiting osteoclast differentiation, maturation, and function. It has demonstrated significant clinical benefit by reducing the overall risk of skeletal-related events (SREs) by approximately 18% in bone metastatic patients (6, 7). Beyond its anti-resorptive role, denosumab also exerts key immunomodulatory functions by blocking RANKL–RANK signaling, including promoting dendritic cell maturation, enhancing CD8^+^ T-cell cytotoxicity, and alleviating the immunosuppressive microenvironment (8–10). In parallel, PD-1/PD-L1 immune checkpoint inhibitors (ICIs) have become the standard first-line therapy for advanced lung cancer, restoring T-cell function and effectively enhancing antitumor immunity (11, 12).

Given their complementary mechanisms, the combination of denosumab and PD-1/PD-L1 ICIs shows promising synergistic effects in the bone metastatic setting, with improved objective response rate (ORR), prolonged progression-free survival (PFS), and manageable safety profiles in lung cancer patients (13, 14). This provides new therapeutic opportunities for this high-risk population.

With the rise of short-video platforms and social media, medical and health-related information has become widely disseminated through algorithm-driven personalized recommendations, making these platforms a major source of health knowledge for the general public (15). However, while recommendation algorithms improve information accessibility, they also reinforce the “algorithmic echo chamber” effect, characterized by content homogenization and strengthened cognitive biases (16). These issues are particularly evident on Chinese platforms such as Xiaohongshu, Bilibili, and Douyin, where complex medical concepts are frequently oversimplified, anecdotal evidence is generalized, individualized treatment principles are overlooked, and essential evidence-based perspectives such as risk communication and adverse effects are insufficiently addressed—even in videos posted by healthcare professionals (17–19). Continuous exposure to selectively amplified content such as “successful miracles” or extreme negative experiences may distort risk perception, reinforce biased interpretations, and influence real-world health cognition and decision-making behavior (20–22).

Meanwhile, advances in natural language processing (NLP) and machine learning have greatly enhanced the objectivity, scalability, and multimodal analytical capacity of short-video quality assessment. AI-based frameworks can process text, audio, and visual features simultaneously, minimizing subjective bias and enabling comprehensive evaluation according to internationally recognized metrics, such as the Global Quality Score (GQS), DISCERN, and JAMA criteria (23–25). These technologies provide a robust foundation for systematic evaluation of online cancer-related information quality.

Despite the emerging evidence supporting the clinical benefits of denosumab combined with PD-1/PD-L1 inhibitors for lung cancer bone metastasis (13), studies investigating how related information is disseminated on short-video platforms remain scarce. Research on communication characteristics, audience cognition, and potential misinformation in the context of cancer immunotherapy is largely lacking (26, 27). Moreover, little is known about how the quality of disseminated information and resultant cognitive biases may influence treatment understanding, adherence behaviors, and ultimately patient outcomes (28). Therefore, it is imperative to establish AI-driven assessment models to systematically evaluate the quality of short-video content and explore how informational differences may shape patient perception and potential immunotherapy decision-making.

**Figure 1:**
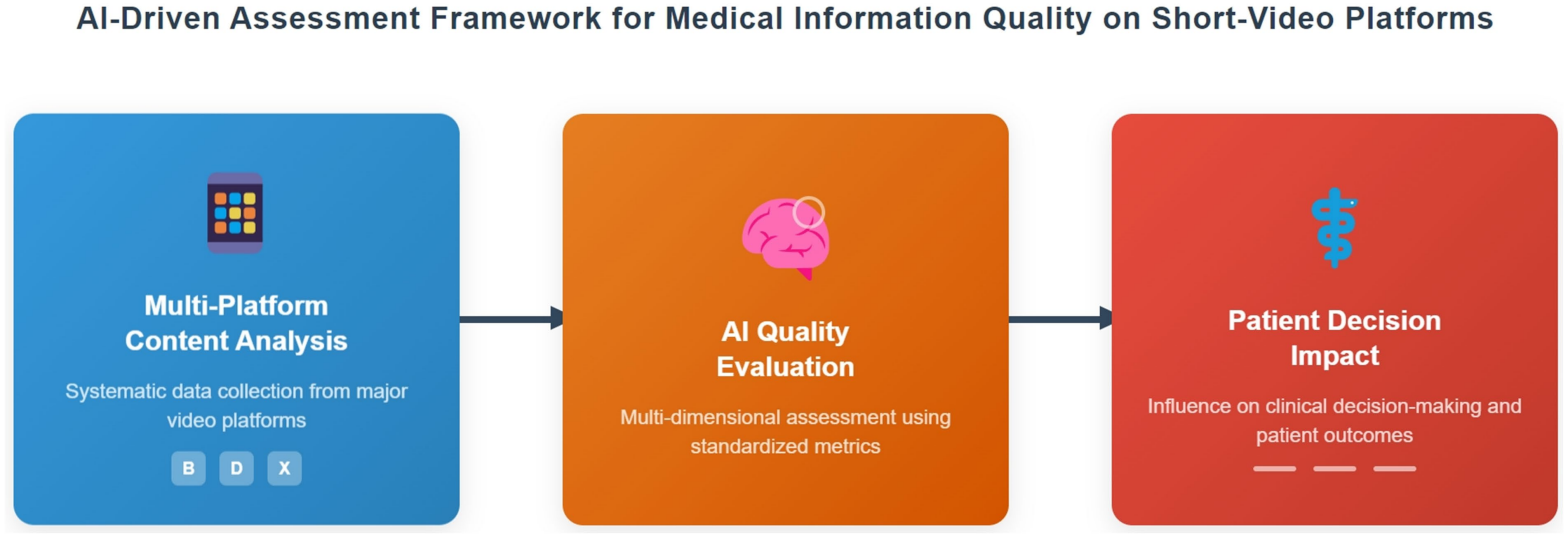
Conceptual framework of AI-driven evaluation of short-video medical information and its impact on patient decision-making. This framework illustrates how multi-platform content analysis feeds into an AI-driven quality assessment pipeline, ultimately influencing patients’ cognitive pathways and decision-making behaviors. It highlights a systematic approach to evaluating the quality of immunotherapy-related medical information and its clinical significance.

In this study, we employed AI-based multimodal analysis to quantify the quality of short-video content related to denosumab combined with PD-1/PD-L1 therapy on Douyin, Bilibili, and Xiaohongshu (24, 29), and to investigate how varying content quality may contribute to cognitive bias and influence immunologic awareness and related clinical decision-making among patients with lung cancer bone metastasis (Figure 1).

## 2 Materials and methods

### 2.1 Data collection

This cross-sectional observational study systematically evaluated the quality of medical short-video content related to “denosumab combined with PD-1/PD-L1 inhibitors for lung cancer bone metastasis” across major Chinese social media platforms. The study adhered to the Strengthening the Reporting of Observational Studies in Epidemiology (STROBE) guidelines to ensure transparency and methodological rigor in observational research (30, 31). Based on user population, content characteristics, and influence on medical science communication, three widely used short-video platforms were selected: Bilibili (the largest video-sharing platform in China, with over 270 million monthly active users), Xiaohongshu (also known as RED, a lifestyle-sharing platform with more than 200 million monthly active users), and Douyin (the Chinese version of TikTok, with over 600 million daily active users) (32, 33).

A unified search strategy was applied using the core keyword phrase “denosumab combined with PD-1/PD-L1 immunotherapy for lung cancer bone metastasis” to conduct a systematic retrieval across the three platforms. Given that Bilibili, Douyin, and Xiaohongshu incorporate intelligent synonym recognition and keyword expansion algorithms, enabling automatic matching with relevant terminologies, the use of a single core search term was considered sufficient for comprehensive dataset coverage and aligned with best practices in current short-video content research (34). Data extraction was performed on August 26, 2025, including all eligible videos available at the time of retrieval. No restrictions were applied regarding the publication date of the videos. A self-developed web crawler was utilized to collect data from the three platforms, yielding a total of 1,369 initial videos.

A standardized manual relevance assessment based on a three-level keyword scoring system was employed for screening (35). Primary keywords (high weight) included *denosumab*, bone metastasis, and lung cancer bone metastasis; secondary keywords (medium weight) covered immunotherapy, PD-1, PD-L1, immune checkpoint inhibitors, lung cancer, and combination therapy; tertiary keywords (low weight) consisted of bone lesions, osteoporosis, tumor therapy, targeted therapy, clinical trials, and evidence-based medicine.

The relevance scoring criteria were as follows: 3 points (high relevance): videos containing both primary and secondary keywords; 2 points (moderate relevance): videos containing primary keywords only; 1 point (low relevance): videos containing only secondary or tertiary keywords;0 point (irrelevant): videos with no related keywords.

Videos with a relevance score ≥1 were included in the preliminary screening. Following the initial screening, manual speech-to-text transcription was performed to supplement content data. Additionally, metadata—including likes, shares, user preference metrics, and video duration—were extracted and completed whenever possible, with the exception of view count data from Xiaohongshu, which is not publicly accessible. Videos with incomplete transcription content were systematically excluded to ensure full textual availability for subsequent analyses.

After de-duplication and quality filtering, 402 videos fulfilled the statistical power requirements for short-video quality assessment research (36), comprising 222 from Bilibili (55.2%), 105 from Douyin (26.1%), and 75 from Xiaohongshu (18.7%).

Inclusion criteria: (1) Content involving denosumab or treatment of lung cancer bone metastasis; (2) Video duration ≥15 s; (3) Chinese-language audio with clear intelligibility; (4) Relevance score ≥1; (5) Complete textual transcription; (6) Complete platform-specific metadata.

Exclusion criteria: (1) Pure advertisements or commercial promotion; (2) Duplicated or re-posted content; (3) Poor or incomprehensible audio quality; (4) Evidently inaccurate or misleading information; (5) Relevance score <1; (6) Missing transcription data; (7) Missing platform-specific metadata.

Additionally, manual verification of all included videos’ titles and publisher accounts was performed to identify potential cross-platform duplication. No systematic instances of the same video being repeatedly included across different platforms were identified. A few videos with similar titles were confirmed to differ in uploader identity, video duration, and presentation format; therefore, they were retained as independent samples.

### 2.2 Video classification

A systematic classification framework based on the professional background and authority of content creators was applied (37). Healthcare professionals in relevant specialties: oncologists, orthopedic surgeons, and other clinicians with direct experience in managing lung cancer bone metastasis, including those officially verified by Douyin; Healthcare professionals in unrelated fields: licensed clinicians, nurses, pharmacists, and other medical personnel without direct involvement in this disease area; Patients and caregivers: individuals diagnosed with the disease, family caregivers, or patient advocates sharing personal disease experiences; Other individuals: general users without medical training, commercial organizations, media accounts, and popular health-science content creators.

An automated identity-verification system was developed to classify uploader profiles. This system applies intelligent recognition based on uploader names and video content, integrates professional background validation via Doubao API calls, and generates a credibility confidence score (0–1 scale) (38, 39). For Douyin-verified accounts, platform-specific optimization modules were implemented to correct and enhance metadata extraction, ensuring consistent and accurate identification.

### 2.3 Quality assessment

A comprehensive video-quality evaluation system was developed using the state-of-the-art doubao-seed-1.6 model. The system adopts a modular architecture composed of two primary components: (1) a core analytical engine and (2) an integrated visualization generator for quality scoring outputs. The design of this evaluation system was informed by the most recently published framework for assessing the quality of health education short videos (2025 edition) (40). In addition, the system incorporates three internationally recognized quality assessment standards for medical information: the Global Quality Score (GQS), the Journal of the American Medical Association (JAMA) benchmark criteria, and the modified DISCERN instrument ((m)DISCERN) (40, 41).

The Global Quality Score (GQS) is a five-point scale developed by Bernard et al. (36), assessing content accuracy, completeness of information, clarity of expression, logical organization, and practical value (17). The JAMA benchmark criteria, proposed by Silberg et al. (42), range from 0 to 4 points and evaluate four standards: authorship, attribution, disclosure of sources, and currency of information. The modified DISCERN ((m)DISCERN) tool, derived from the original DISCERN instrument developed by Charnock et al. (43), is one of the most frequently used instruments in health-information research. It employs a 5-point scoring system covering treatment options, risk–benefit evaluation, information quality, decision-support capacity, and overall reliability.

Video quality assessment was first conducted using the doubao-seed-1.6 large language model with a structured prompt template designed on the basis of medical domain knowledge. The system incorporates an intelligent speech-to-text module capable of correcting medical terminology errors, including homophones, and accurately mapping drug names, diagnoses, and treatment-related vocabulary. An automated identity-recognition component analyzes uploader names, institutional affiliations, and video content to detect keywords related to authoritative medical organizations and professional credentials. This enables a multidimensional framework for assessing the credibility of uploaders and represents an advanced technical approach in short-video quality evaluation (44).

Instead of traditional inter-rater reliability testing, this study adopted a Quality Management System (QMS) approach to ensure the professionalism and accuracy of evaluations (45, 46). The quality assessment procedures consisted of four stages:

(1) AI preliminary scoring: Each video was independently evaluated three times by the doubao-seed-1.6 large language model. A re-evaluation process was automatically triggered when the score difference exceeded 1 point, continuing until consistent results were obtained.
(2) Primary expert review: Two clinical specialists in bone oncology independently reviewed AI-generated scores for all 402 videos, evaluating the accuracy and appropriateness of the scoring based on GQS, JAMA, and mDISCERN standards, with adjustments made if necessary.
(3) Secondary arbitration: When the discrepancy between the two experts exceeded 2 points, arbitration was conducted by a third attending physician certified in bone oncology.
(4) Tertiary quality supervision: A fully blinded supervisory audit was conducted by an independent senior specialist who randomly inspected 10% of the sample (*n* = 40) without access to prior evaluations. The supervisor confirmed that the reviewed scores met the established standards, verifying the reliability and effective execution of the assessment workflow.

This quality management design reflects an ISO-9001–style quality control framework (47), combining complete inspection with random auditing to ensure scoring integrity—providing more comprehensive quality assurance than conventional sampling-based reliability testing.

Additional quality control measures included automated detection and handling of scoring anomalies. Full-process logging captured timestamps, API request parameters, response outputs, AI preliminary scores, expert revision trajectories, and reasons for adjustments. System monitoring encompassed API success rate, response latency, and scoring consistency. A data protection module ensured secure handling of sensitive information through de-identification, access control, encrypted transmission, and operational log tracking.

Comprehensive quality scores were calculated using the composite indicator methodology recommended by the OECD (2008) (39).


$$\begin{array}{l} \text{Overall Score}=0.4\times {\mathrm{GQS}}_{\text{normalized}}+0.3\times {\text{JAMA}}_{\text{normalized}}\\ +0.3\times {\text{DISCERN}}_{\text{normalized}} \end{array}$$

Normative weighting was applied based on expert consensus regarding the relative importance of quality dimensions in digital health information assessments: overall quality and accuracy (GQS) were prioritized with the highest weight (0.4), while source transparency (JAMA) and treatment reliability (mDISCERN) were assigned equal weights (0.3) (48). Similar weighting approaches have been widely adopted in previous studies on online health-information quality (49). To ensure interpretability, individual component scores were reported alongside the overall score.

### 2.4 Statistical analysis

Data processing and statistical analyses were performed using Python version 3.9 or above, and figures were generated in accordance with academic publishing standards (50). Descriptive statistics included measures of central tendency, dispersion, and distribution, stratified by platform. Video content was analyzed using natural language processing techniques for topic classification and keyword extraction (51).

A significance level of *α* = 0.05 was adopted for inferential analyses. Normality was assessed using the Shapiro–Wilk and Jarque–Bera tests. The Kruskal–Wallis test (for multiple groups) or Mann–Whitney U test (for pairwise comparisons) was used for continuous variables, while categorical variables were compared using the Chi-square test (17). Correlation analyses utilized Pearson or Spearman coefficients depending on distributional characteristics, with Kendall’s tau calculated as a robustness check.

Multiple comparison correction was performed using the Benjamini–Hochberg false discovery rate (FDR) method, with Bonferroni correction also provided for comparison (52). Effect sizes were estimated using R^2^ for correlation analyses and Cohen’s d for group comparisons (53).

Multivariable linear regression models were constructed, accompanied by diagnostic evaluations including residual normality, heteroscedasticity, multicollinearity (variance inflation factor >10), and influential point detection (Cook’s distance > 4/n). All analyses were two-tailed, and *p* < 0.05 was considered statistically significant (52, 53).

### 2.5 Model selection

The doubao-seed-1.6 model (Volcengine Ark) was selected as the auxiliary scoring tool for text analysis due to its strong semantic understanding and structured output capabilities in the Chinese language environment, making it suitable for large-scale processing of Chinese health-related video content (official documentation available from Volcengine). However, the research design, analytical interpretation, and manuscript writing were entirely conducted by the authors without the assistance of any generative AI tools.

**Figure 2:**
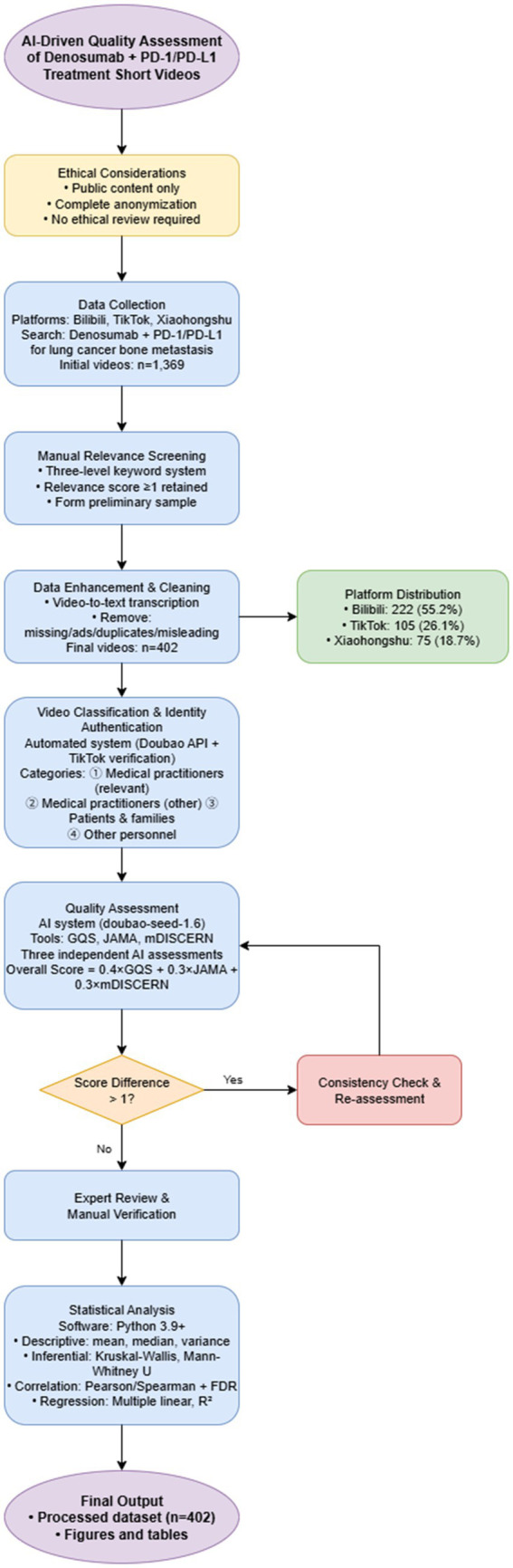
Data processing workflow. Visualization of the data collection, cleaning, extraction, AI-driven scoring, and statistical analysis pipeline for the included short-video dataset.

Figure 2 illustrates the overall data processing workflow. Detailed algorithmic parameters, software configurations, computational formulas, prompt templates, and additional datasets are provided in the Supplementary materials.

## 3 Results

### 3.1 Baseline characteristics and platform distribution

**Figure 3:**
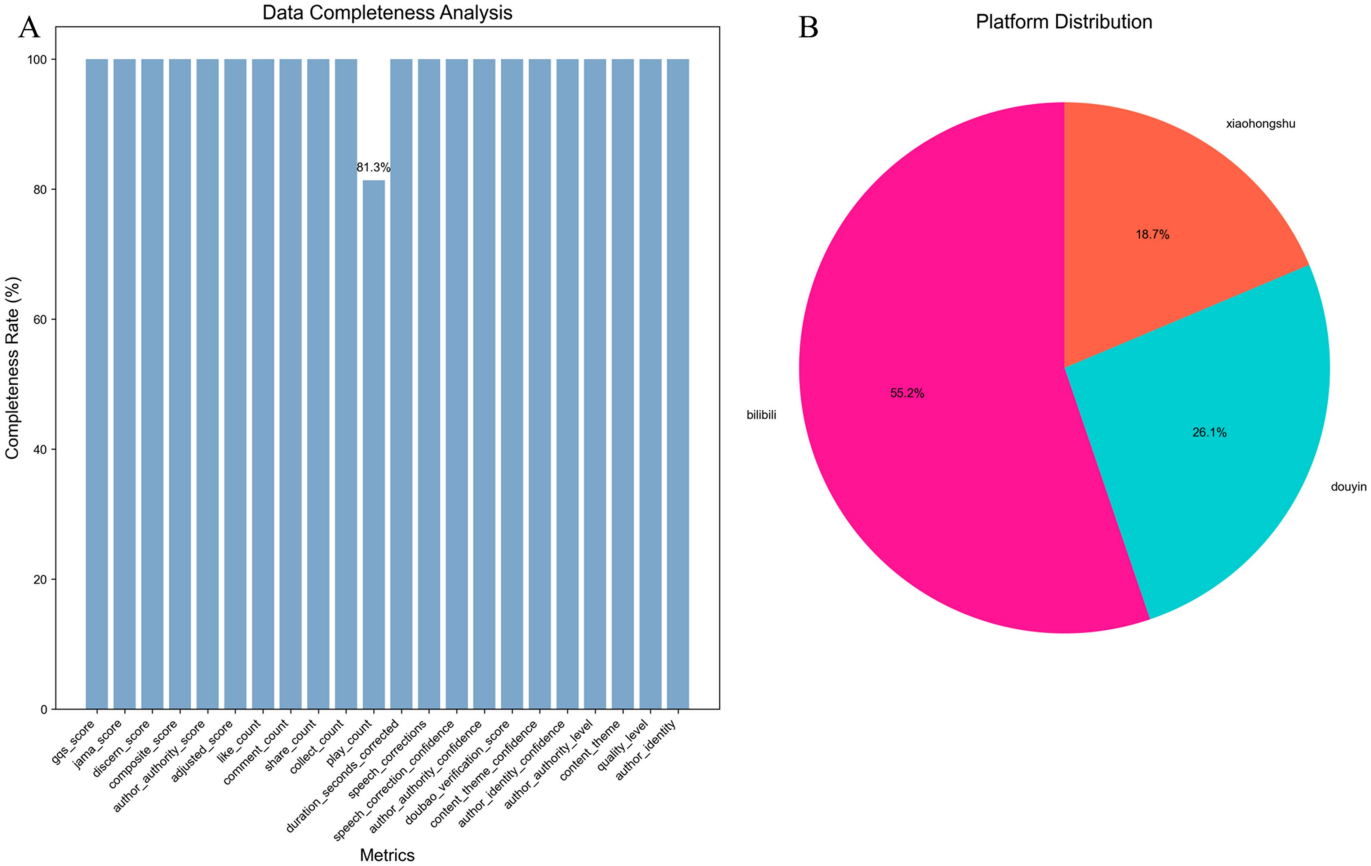
Data completeness and platform distribution of included videos. **(A)** Data completeness across 21 evaluation indicators among 402 videos collected up to August 26, 2025. Indicators include quality scoring (GQS, JAMA, DISCERN, and composite score), content characteristics (author authority score, adjusted score, authority category, content theme, quality grade, creator identity), engagement metrics (likes, comments, shares, favorites, plays), and technical parameters (adjusted duration, speech correction count, speech confidence, creator verification score, Doubao topic confidence, content identity confidence). All indicators except play count achieved 100% completeness; play count completeness was 81.3%. **(B)** Platform distribution showing proportions of Bilibili (55.2%, *n* = 222), Douyin (26.1%, *n* = 105), and Xiaohongshu (18.7%, *n* = 75). All analyses were performed in Python 3.9+.

A total of 402 videos were included in the final analysis, with uneven distribution across platforms (Figure 3): Bilibili accounted for 55.2% (*n* = 222), Douyin 26.1% (*n* = 105), and Xiaohongshu 18.7% (*n* = 75) (Figure 3B). Play count availability was 81.3%, while all remaining 20 evaluation indicators achieved 100% completeness (Figure 3A), ensuring analytical robustness.

Baseline characteristics revealed pronounced platform-level heterogeneity (Table 1). In terms of content quality, significant differences were observed in GQS (2.45 ± 1.03 vs. 3.40 ± 0.85 vs. 3.17 ± 0.92, *p* < 0.001), JAMA (0.25 ± 0.51 vs. 0.50 ± 0.62 vs. 0.41 ± 0.62, *p* < 0.001), and DISCERN scores (1.33 ± 0.61 vs. 1.90 ± 0.67 vs. 1.72 ± 0.69, *p* < 0.001).

**Table 1:** Baseline characteristics of the videos on Bilibili, Douyin, and Xiaohongshu.

Characteristics	Overall (*n* = 402)	Bilibili (*n* = 222)	Douyin (*n* = 105)	Xiaohongshu (*n* = 75)	*p*-value
GQS score	2.84 ± 1.06	2.45 ± 1.03	3.40 ± 0.85	3.17 ± 0.92	<0.001 *
JAMA score	0.34 ± 0.57	0.25 ± 0.51	0.50 ± 0.62	0.41 ± 0.62	<0.001 *
DISCERN score	1.55 ± 0.69	1.33 ± 0.61	1.90 ± 0.67	1.72 ± 0.69	<0.001 *
Likes	56.50 (0, 36,939)	26.50 (0, 36,939)	207.00 (5, 5,619)	33.00 (2, 493)	<0.001 *
Shares	11.00 (0, 3,275)	4.00 (0, 2,363)	57.00 (0, 3,275)	17.00 (0, 365)	<0.001 *
Comments	5.50 (0, 2,849)	2.00 (0, 2,849)	18.00 (0, 738)	3.00 (0, 130)	<0.001 *
Collects	23.00 (0, 5,870)	13.00 (0, 3,703)	92.00 (0, 5,870)	28.00 (0, 451)	<0.001 *
Play count	32,079.45 ± 75,610.26	13,120.52 ± 41,700.56	72,164.04 ± 108,791.49	N/A	<0.001 *
Duration (sec)	100.50 (18, 435,420)	127.50 (18, 435,420)	83.00 (18, 386)	67.00 (19, 414)	<0.001 *

The table presents the baseline characteristics of videos across three platforms (Bilibili, Douyin, and Xiaohongshu). Quality scores (GQS, JAMA, and DISCERN) are reported as mean ± SD, while engagement metrics (Likes, Shares, Comments, Collects) and video duration are presented as median (min, max). Play count data is provided as mean ± SD. The statistical significance of differences between platforms was assessed using the Kruskal–Wallis test, with all comparisons showing *p*-values less than 0.001. Play count data for Xiaohongshu were not publicly available. Outlier Note: The maximum duration on Bilibili reached 435,420 s (~121.5 h), indicating the presence of long-form or archival videos, in contrast to short-form norms on Douyin and Xiaohongshu.

**Figure 4:**
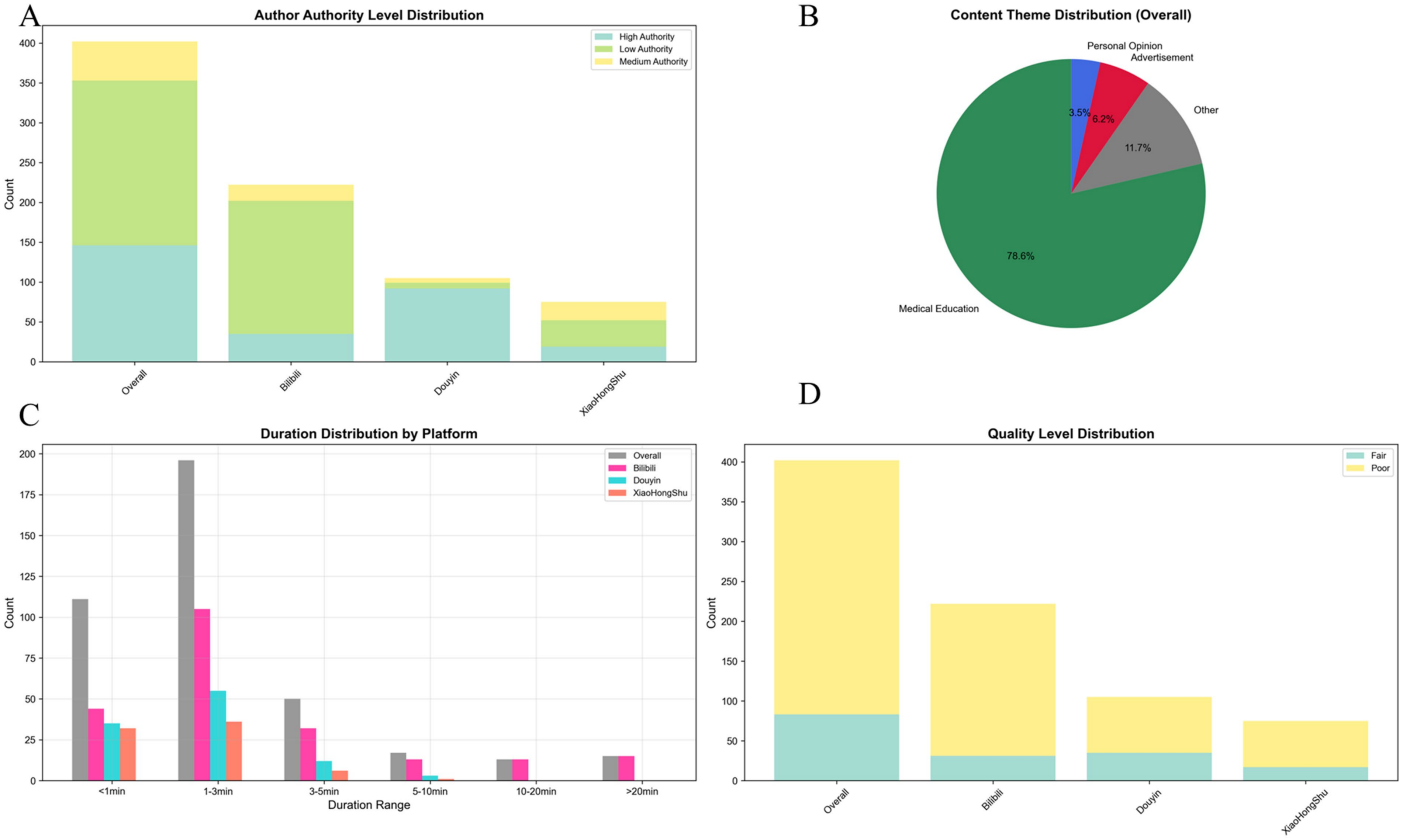
Multidimensional distribution of short-video content characteristics. **(A)** Distribution of creator authority levels across platforms. Authority level was derived from a weighted scoring system including identity (40%), verification status (30%), professional qualifications (20%), and AI-supported validation (10%). **(B)** Content theme distribution across all videos: medical education (78.6%), advertisements (6.2%), personal opinions (3.5%), and others (11.7%). **(C)** Platform-specific distribution of video duration grouped into <1 min, 1–3 min, 3–5 min, 5–10 min, 10–20 min, and >20 min categories. **(D)** Quality grade distribution based on the composite scoring system integrating GQS (40%), JAMA (30%), and revised DISCERN (30%). Only 0.2% (*n* = 1) were rated “good,” 19.2% (*n* = 77) “fair,” and 80.6% (*n* = 324) “poor,” with no videos achieving “excellent”.

User engagement metrics displayed similar variation: Douyin had a significantly higher median “likes” count (207) than Bilibili (26.5) and Xiaohongshu (33) (*p* < 0.001), with consistent trends in shares, comments, and favorites. Video duration also differed significantly, with Bilibili showing the longest median length (127.5 seconds), followed by Douyin (83 seconds) and Xiaohongshu (67 seconds) (*p* < 0.001; Figure 4C).

These findings imply intrinsic platform differences in content ecosystems, user engagement patterns, and algorithmic dissemination mechanisms.

### 3.2 Multidimensional evaluation of video content quality

Overall, the included videos exhibited notably low quality relative to established medical information standards. The quality grade distribution was severely skewed: 80.6% (*n* = 324) were classified as “poor,” 19.2% (*n* = 77) as “fair,” only 0.2% (*n* = 1) as “good,” and none reached the “excellent” threshold (Figure 4D).

**Figure 5:**
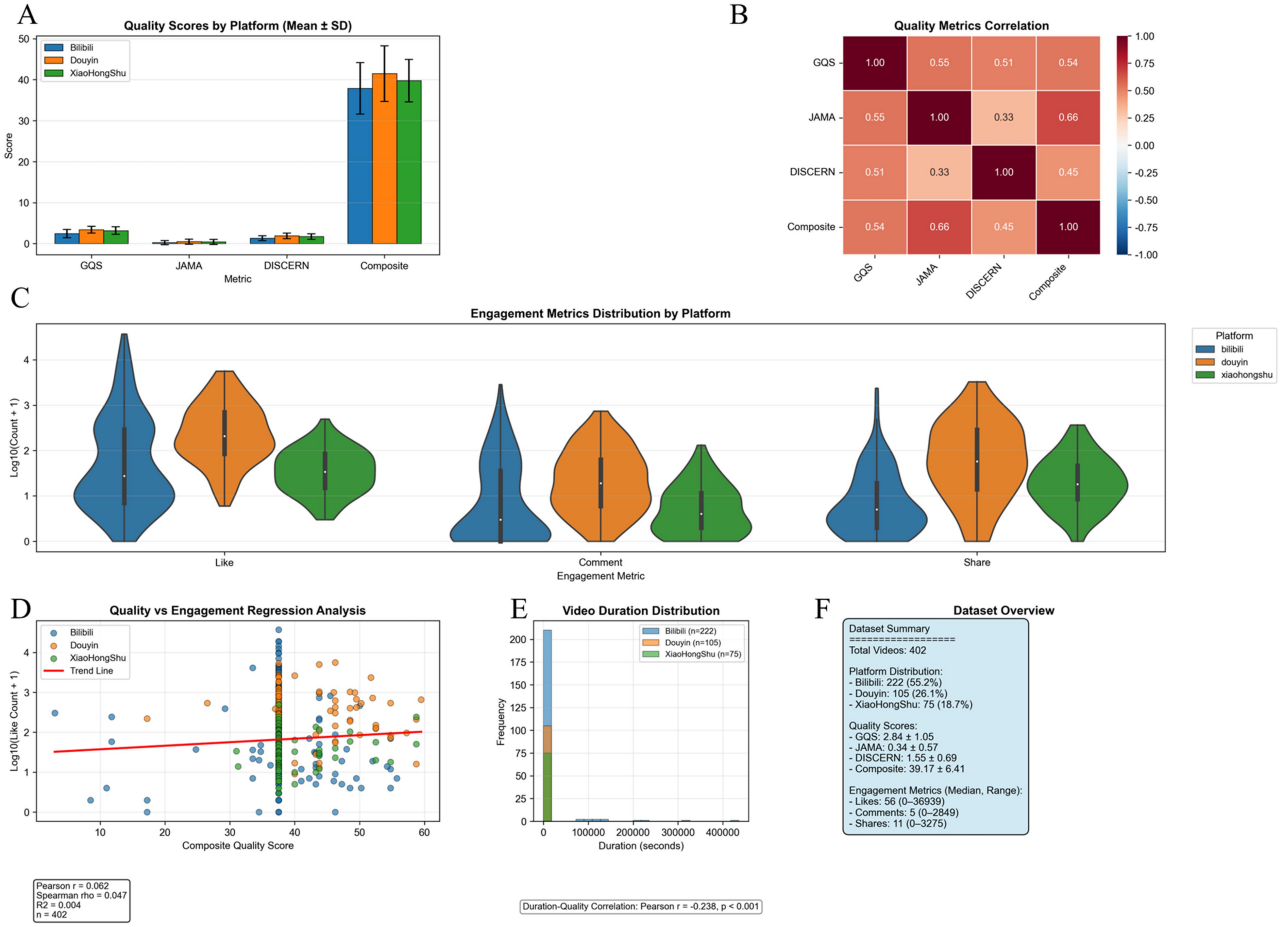
Platform differences in multidimensional quality assessments and associations with engagement. **(A)** Mean ± SD comparison of quality indicators (GQS, JAMA, DISCERN, composite score) across platforms using Kruskal–Wallis test (all *p* < 0.001). **(B)** Pearson correlation matrix of quality indicators showing moderate-to-strong positive correlations among scoring systems. **(C)** Violin plots of log10(x + 1)-transformed engagement metrics (likes, comments, shares) showing distinct distribution patterns across platforms. **(D)** Scatter plots with fitted regression lines showing a weak association between composite score and log10-transformed likes (Pearson r = 0.062; Spearman *ρ* = 0.047; R^2^ = 0.004; *n* = 402). **(E)** Histogram of video duration indicating right-skewed distribution and weak negative correlation with composite score (Pearson r = −0.238; *p* < 0.001). **(F)** Summary panel presenting overall dataset characteristics: platform distribution, mean quality scores (GQS 2.84 ± 1.05; JAMA 0.34 ± 0.57; DISCERN 1.55 ± 0.69; composite 39.17 ± 6.41), and median engagement metrics (likes 56, comments 5, shares 11).

**Figure 6:**
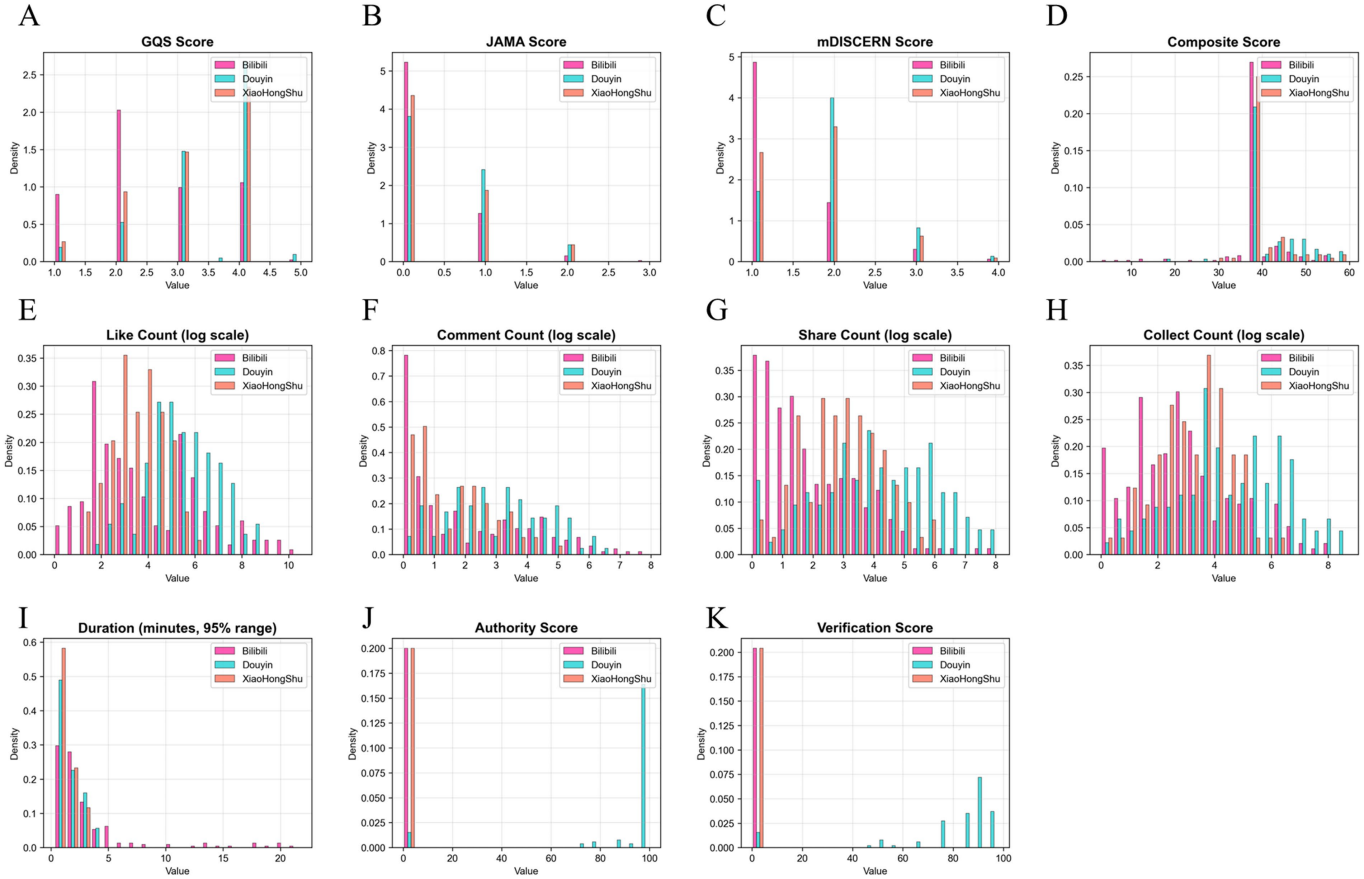
Distribution of quality, engagement, and content characteristics across short-video platforms. Upper panel (Quality indicators): Histograms show the platform-specific distributions of four quality indicators. **(A)** GQS (1–5 scale): Bilibili (2.455 ± 1.031, median = 2.000, *n* = 222), Douyin (3.398 ± 0.849, median = 4.000, *n* = 105), Xiaohongshu (3.173 ± 0.921, median = 3.000, *n* = 75); Kruskal–Wallis H = 66.805, *p* < 0.000001. **(B)** JAMA (0–4 scale): Bilibili (0.248 ± 0.510), Douyin (0.495 ± 0.622), Xiaohongshu (0.413 ± 0.617); H = 16.720, *p* = 0.000234. **(C)** Modified DISCERN (1–5 scale): Bilibili (1.333 ± 0.607), Douyin (1.905 ± 0.764), Xiaohongshu (1.653 ± 0.714); H = 37.901, *p* < 0.000001. **(D)** Composite quality score: Bilibili (constant 37.500 ± 0.000), Douyin (41.474 ± 6.830; range 17.250–59.500), Xiaohongshu (39.757 ± 5.240; range 31.000–58.750); H = 25.798, *p* = 0.000003. Middle panel (Engagement indicators, log-transformed): Data were transformed using log10(1 + x) to correct skewness. **(E)** Likes: Bilibili (0–36,939), Douyin (5–5,619), Xiaohongshu (0–1,700). **(F)** Comments: Bilibili (0–3,703), Douyin (0–5,870), Xiaohongshu (0–451). **(G,H)** Shares and favorites exhibit similar platform-dependent patterns. All engagement indicators showed significant platform differences (all *p* < 0.000001). Lower panel (Content characteristics): **(I)** Video duration (minutes): extreme values above the 95th percentile removed for clarity. **(J)** Authority score (0–100): platform-specific availability; most Bilibili and Xiaohongshu videos ≈0 due to absence of authority-scoring systems. **(K)** Verification score (0–100): only present on Douyin (78.581 ± 25.315, median = 90.000; 0–98.000); Bilibili and Xiaohongshu values are uniformly zero due to lack of verification mechanisms.

Across specific quality indicators, the overall mean GQS score was 2.84 ± 1.06 (out of 5; pass rate: 56.8%), with significant inter-platform differences (Kruskal–Wallis H = 66.805, *p* < 0.001) (Figures 5A, 6A). JAMA performance was even weaker, with a mean of 0.34 ± 0.57 (out of 4; pass rate: 8.5%), and the highest platform (Douyin) scored only 0.50 ± 0.62 (Figure 6B). DISCERN scores were similarly low (mean 1.55 ± 0.69; pass rate: 31.0%), again with significant variations among platforms (H = 37.901, *p* < 0.001) (Figure 6C).

Internal consistency among quality measures showed moderate correlations: GQS vs. JAMA: r = 0.55, *p* < 0.001; GQS vs. DISCERN: r = 0.51, *p* < 0.001; JAMA vs. composite score: r = 0.66, *p* < 0.001 (Figure 5B), indicating that these tools captured complementary aspects of video quality.

### 3.3 Characteristics and authority of content creators

**Figure 7:**
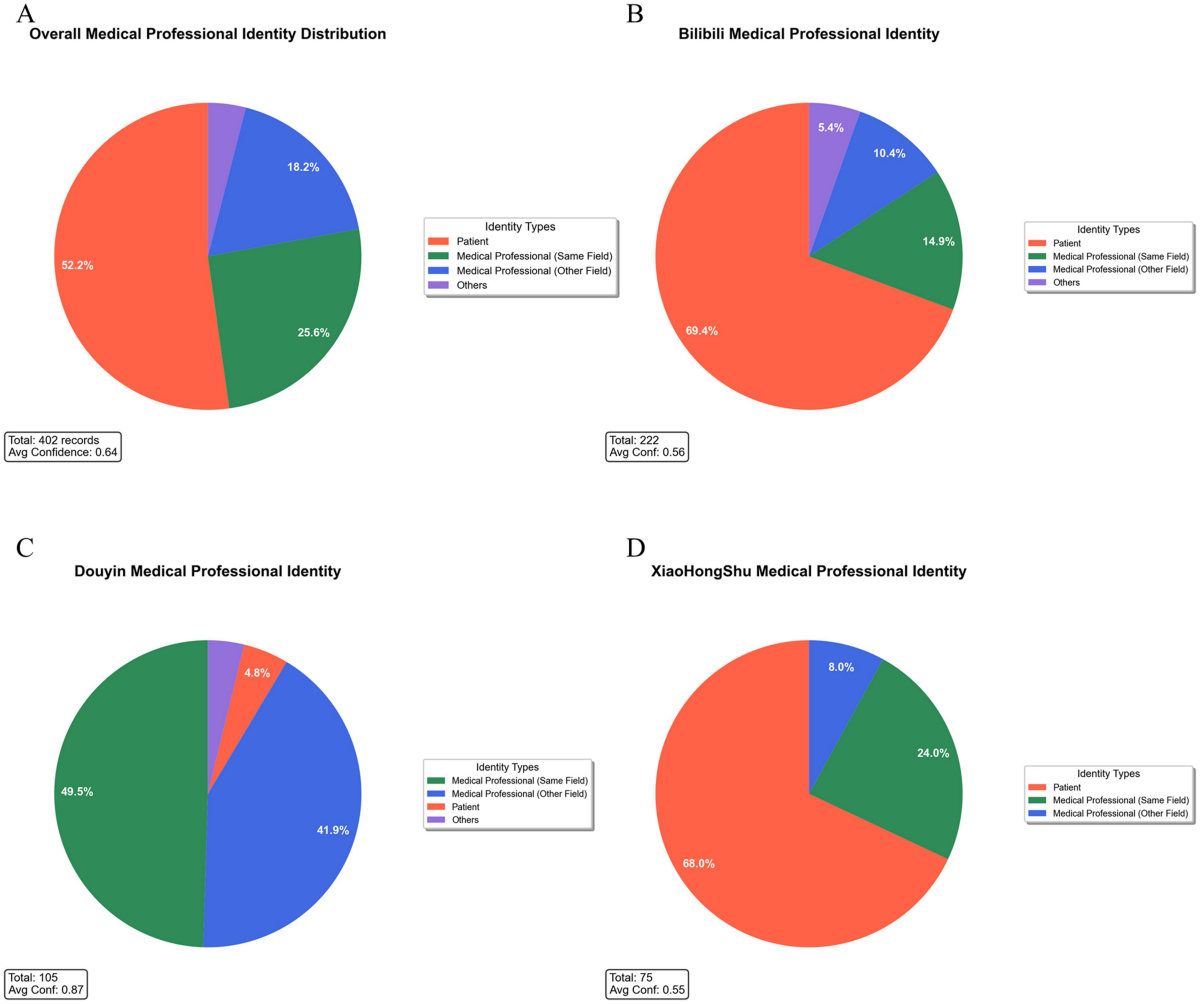
Distribution of creator medical expertise across platforms. **(A)** Overall identity distribution among 402 videos: patient/caregiver-generated content dominated (52.2%, *n* = 210), followed by same-field professionals (25.6%, *n* = 103), other medical fields (18.2%, *n* = 73), and others (4.0%, *n* = 16). Mean confidence score for identity recognition: 0.64. **(B)** Bilibili (*n* = 222): patient content most prevalent (69.4%), lower identity confidence (0.56). **(C)** Douyin (*n* = 105): dominated by same-field professionals (49.5%) and other medical professionals (41.9%); high confidence score (0.87). **(D)** Xiaohongshu (*n* = 75): patient content remains dominant (68.0%); moderate identity confidence (0.55). These findings reveal substantial platform heterogeneity in professional participation and identification certainty.

Analysis of content creators highlighted a substantial deficit in professional medical involvement. Among the 402 videos, 52.2% (*n* = 210) were produced by patients or caregivers; only 25.6% (*n* = 103) originated from healthcare professionals in the relevant field; 18.2% (*n* = 73) from other medical disciplines; and 4.0% (*n* = 16) from other creator categories (Figure 7A). Platform-specific distributions differed significantly: Bilibili had the highest proportion of patient-generated content (69.4%), whereas Douyin demonstrated higher professional engagement (relevant field 49.5%; other medical fields 41.9%), with Xiaohongshu showing intermediate values (Figures 7B–D).

**Figure 8:**
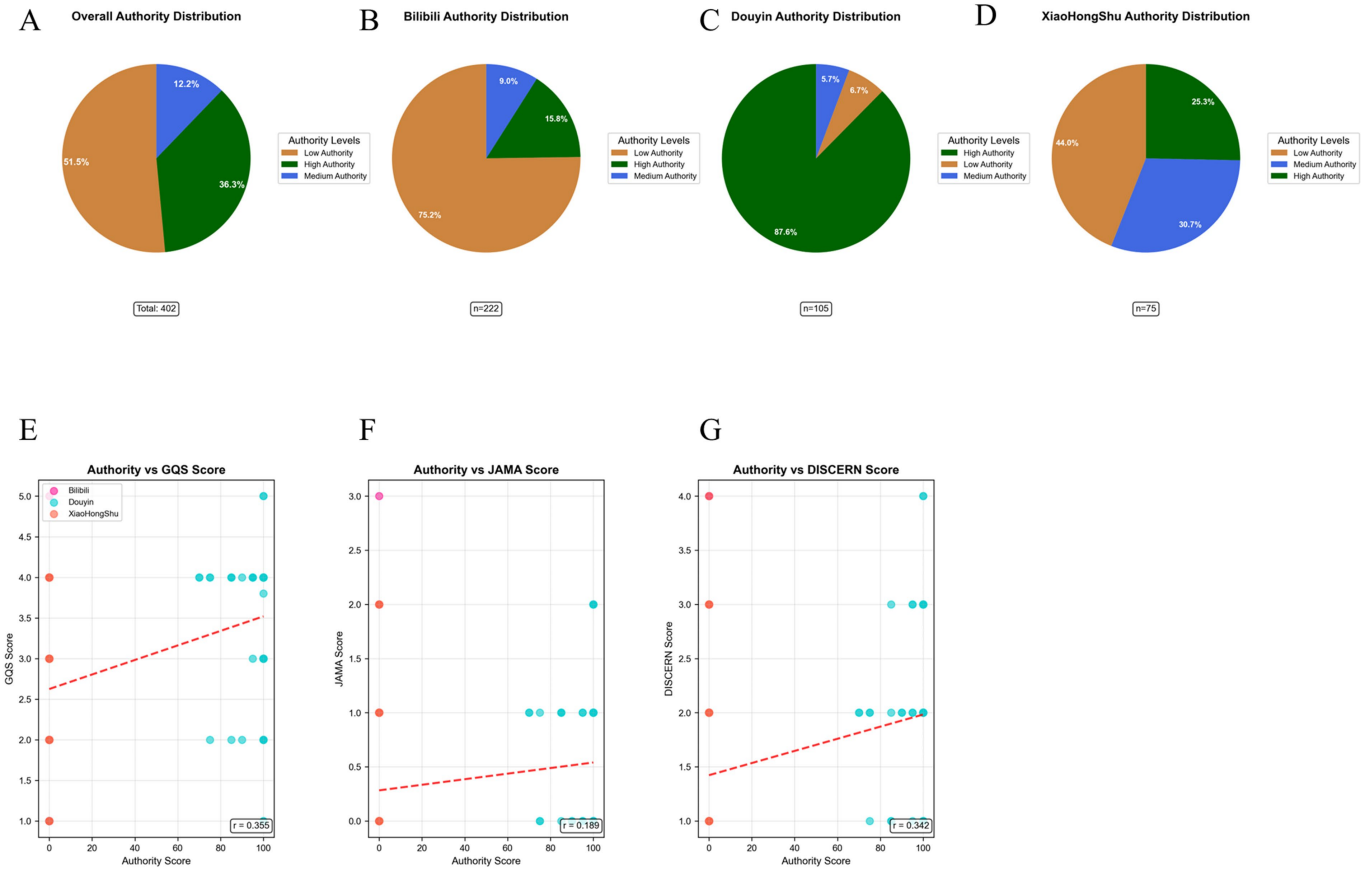
Comprehensive analysis of content creator authority levels across short-video platforms. Upper panels **(A–D)**: Pie charts depict the distribution of creator authority levels for videos discussing denosumab combined with PD-1/PD-L1 inhibitors across four datasets: overall (*n* = 402), Bilibili (*n* = 222), Douyin (*n* = 105), and Xiaohongshu (*n* = 75). Authority levels were classified into three tiers: High authority (green): certified medical professionals with verified clinical credentials; Medium authority (blue): healthcare-associated professionals or medical students with partial medical qualifications; Low authority (orange): non-professional creators including patients, caregivers, and the general public. Authority categorization was based on creator profile information, professional credential verification, and self-reported expertise extracted by the Doubao AI model (doubao-seed-1.6). Lower panels **(E–G)**: Scatter plots with linear regression lines illustrate the relationships between creator authority scores (x-axis, 0–100 scale) and three international health information quality indices (y-axis): Global Quality Score (GQS; 1–5), JAMA benchmark score (0–4), and DISCERN score (1–5). Individual points represent single videos and are color-coded by platform (Bilibili: red; Douyin: blue; Xiaohongshu: pink). Spearman correlation coefficients indicate weak-to-moderate positive associations: GQS: r = 0.355, *p* < 0.001; JAMA: r = 0.189, *p* < 0.001; DISCERN: r = 0.342, *p* < 0.001. These findings suggest that higher creator authority is statistically associated with better information quality, yet the strength of association remains limited, reflecting the persistent presence of low-quality content even among videos with professional creators.

Authority assessment further revealed that high-authority creators (certified medical professionals) accounted for only 36.3%, moderate-authority creators for 12.2%, and low-authority creators for 51.5% (Figures 4A, 8A–D). Authority level exhibited weak-to-moderate positive correlation with quality metrics: GQS: r = 0.355, *p* < 0.001; JAMA: r = 0.189, *p* < 0.001; DISCERN: r = 0.342, *p* < 0.001 (Figures 8E–G).

**Figure 9:**
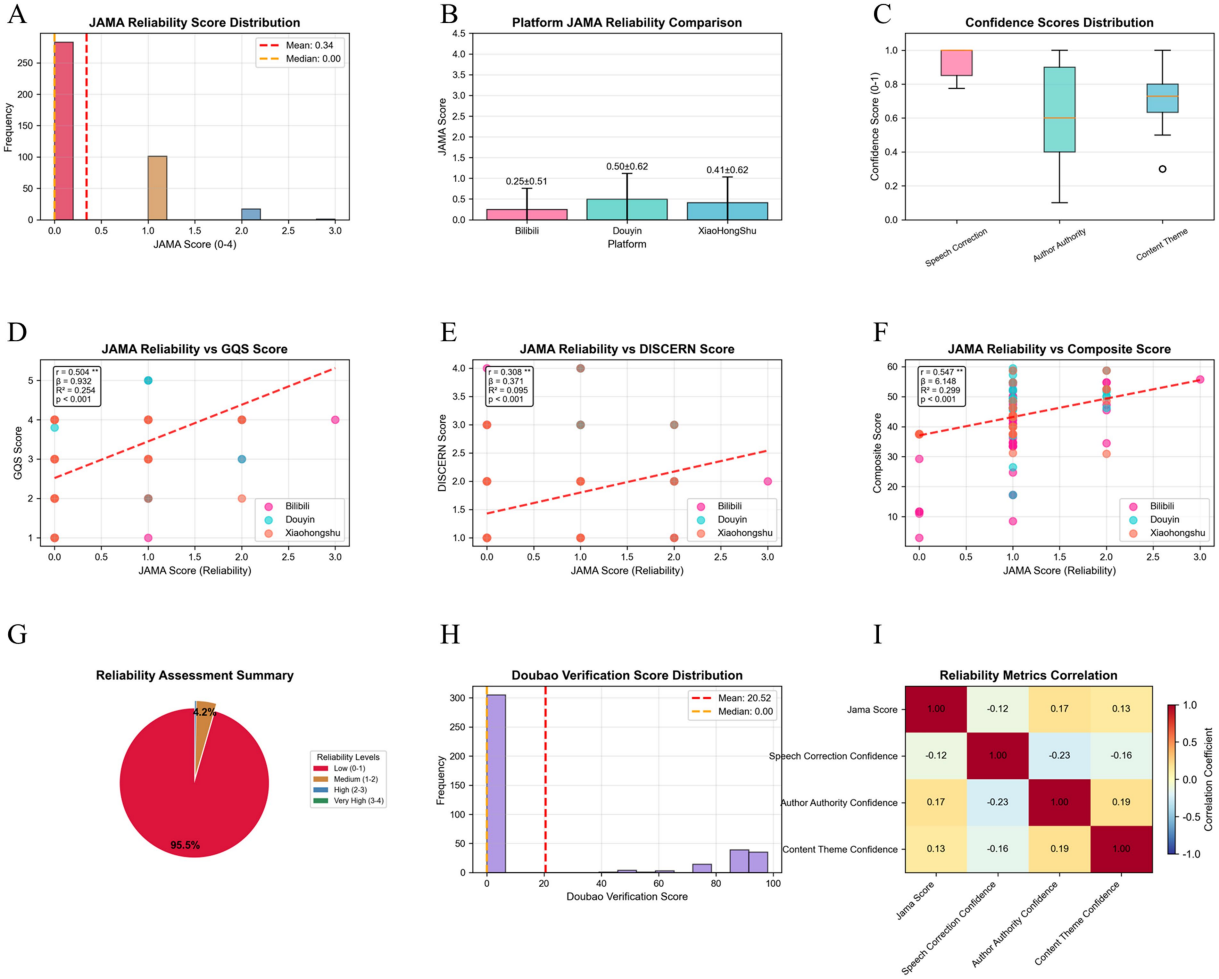
Multidimensional reliability analysis of video content across platforms. Upper panel **(A)** Histogram of JAMA reliability scores (0–3) across 402 videos: 95.5% (*n* = 384) fall within 0–1 range, indicating extremely low reliability. **(B)** Box plots comparing JAMA scores across platforms: Kruskal–Wallis H = 16.720, *p* = 0.000234. **(C)** Box plots of three confidence indicators: speech confidence (0.940 ± 0.068), authority confidence (0.639 ± 0.273), topic confidence (0.682 ± 0.162). Middle panel: Regression analysis of JAMA vs. **(D)** GQS (r = 0.504/ρ = 0.546; *p* < 0.001) **(E)** DISCERN (r = 0.308/ρ = 0.326; *p* < 0.001) **(F)** Composite score (r = 0.547/ρ = 0.657; *p* < 0.001) Platform represented with distinct colors. Lower panel **(G)** Pie chart summarizing JAMA-based reliability categories: low 95.5%, moderate 4.2%, high 0.2%, very high 0%. **(H)** Histogram of Doubao verification scores: mean 20.525 ± 36.889, median 0.000; 92.4% of non-zero values from Douyin only. **(I)** Correlation heatmap: speech confidence negatively correlates with other measures (−0.124 to −0.227), while JAMA positively correlates with authority/topic confidence (0.131–0.174).

Reliability ratings based on JAMA criteria showed that 95.5% (*n* = 384) were classified as low reliability (0–1 points), 4.2% (*n* = 17) as moderate (1–2 points), only 0.2% (*n* = 1) as high (2–3 points), and none achieved a very high rating (Figures 9A,G). Although differences existed across platforms (H = 16.720, *p* = 0.000234), even Douyin (0.495 ± 0.622) fell well below acceptable reliability thresholds (Figure 9B).

### 3.4 Characteristics and topic analysis of video content

**Figure 10:**
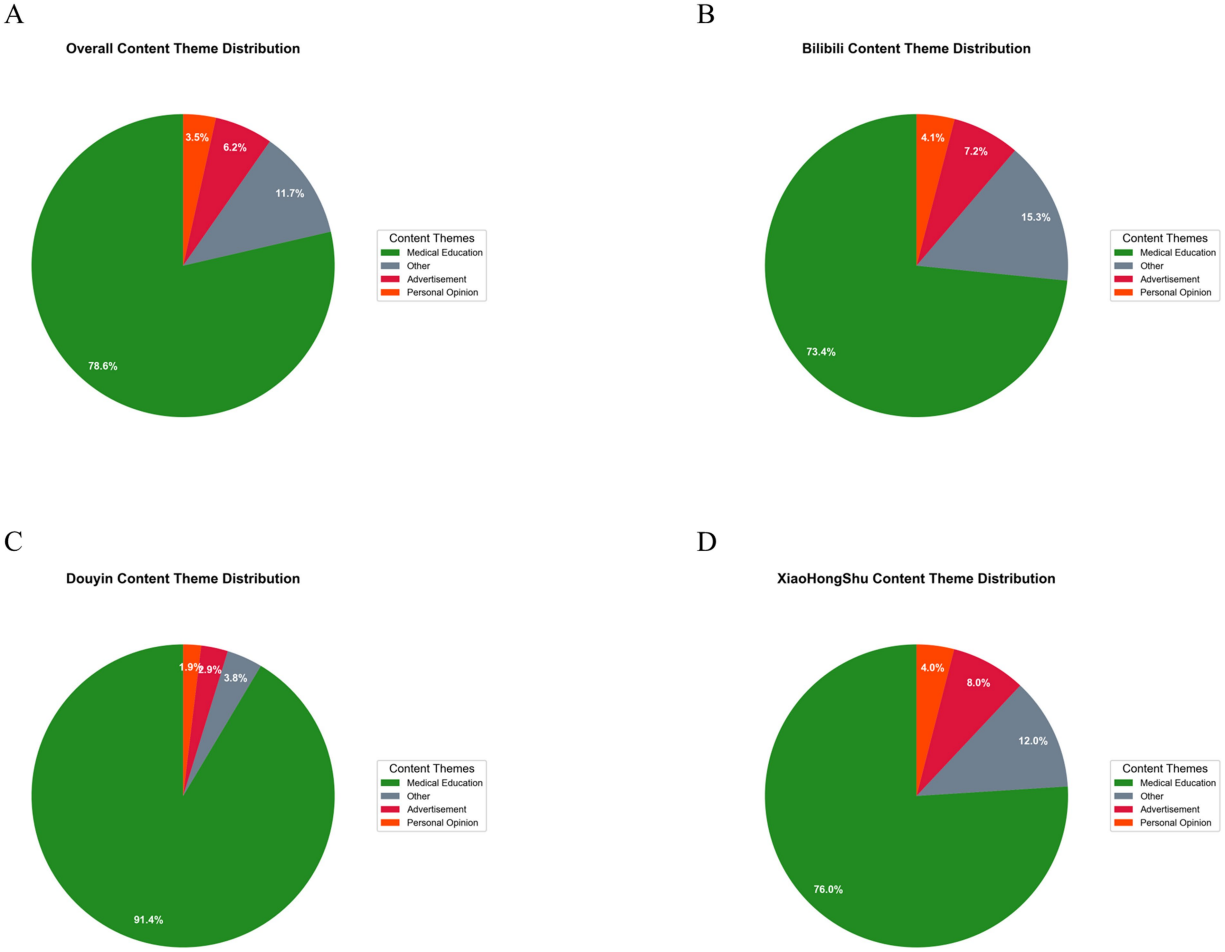
Distribution of video content themes across platforms. **(A)** Overall distribution of content themes among all 402 included videos related to denosumab combined with PD-1/PD-L1 inhibitors for lung cancer bone metastases. Medical education content predominates (78.6%, *n* = 316), followed by other content (11.7%, *n* = 47), advertisements (6.2%, *n* = 25), and personal opinion videos (3.5%, *n* = 14), indicating that health education and popular science are the primary focus across platforms. **(B)** Theme distribution on Bilibili (*n* = 222). Medical education remains the main category (73.4%, *n* = 163), with a higher proportion of “other” content (15.3%, *n* = 34), advertisements (7.2%, *n* = 16), and personal opinions (4.1%, *n* = 9), reflecting slightly lower educational dominance but greater thematic diversity. **(C)** Theme distribution on Douyin (*n* = 105). Medical education content accounts for the highest proportion across platforms (91.4%, *n* = 96), while other content (3.8%, *n* = 4), advertisements (2.9%, *n* = 3), and personal opinions (1.9%, *n* = 2) are relatively rare, indicating a strong educational orientation but limited content diversity. **(D)** Theme distribution on Xiaohongshu (*n* = 75). Medical education comprises 76.0% (*n* = 57), followed by other content (12.0%, *n* = 9), advertisements (8.0%, *n* = 6), and personal opinions (4.0%, *n* = 3), showing a balanced pattern with moderate thematic diversity.

The majority of included videos were categorized as medical education content (78.6%, *n* = 316), although substantial gaps in quality and professional accuracy were observed. The remaining videos included miscellaneous content (11.7%), advertisements (6.2%), and personal opinions (3.5%) (Figure 4B; Figure 10A). Douyin showed the highest proportion of medical education content (91.4%), whereas Bilibili demonstrated greater heterogeneity across content types (73.4% medical education, 15.3% miscellaneous), with Xiaohongshu displaying intermediate characteristics (Figures 10B–D).

**Figure 11:**
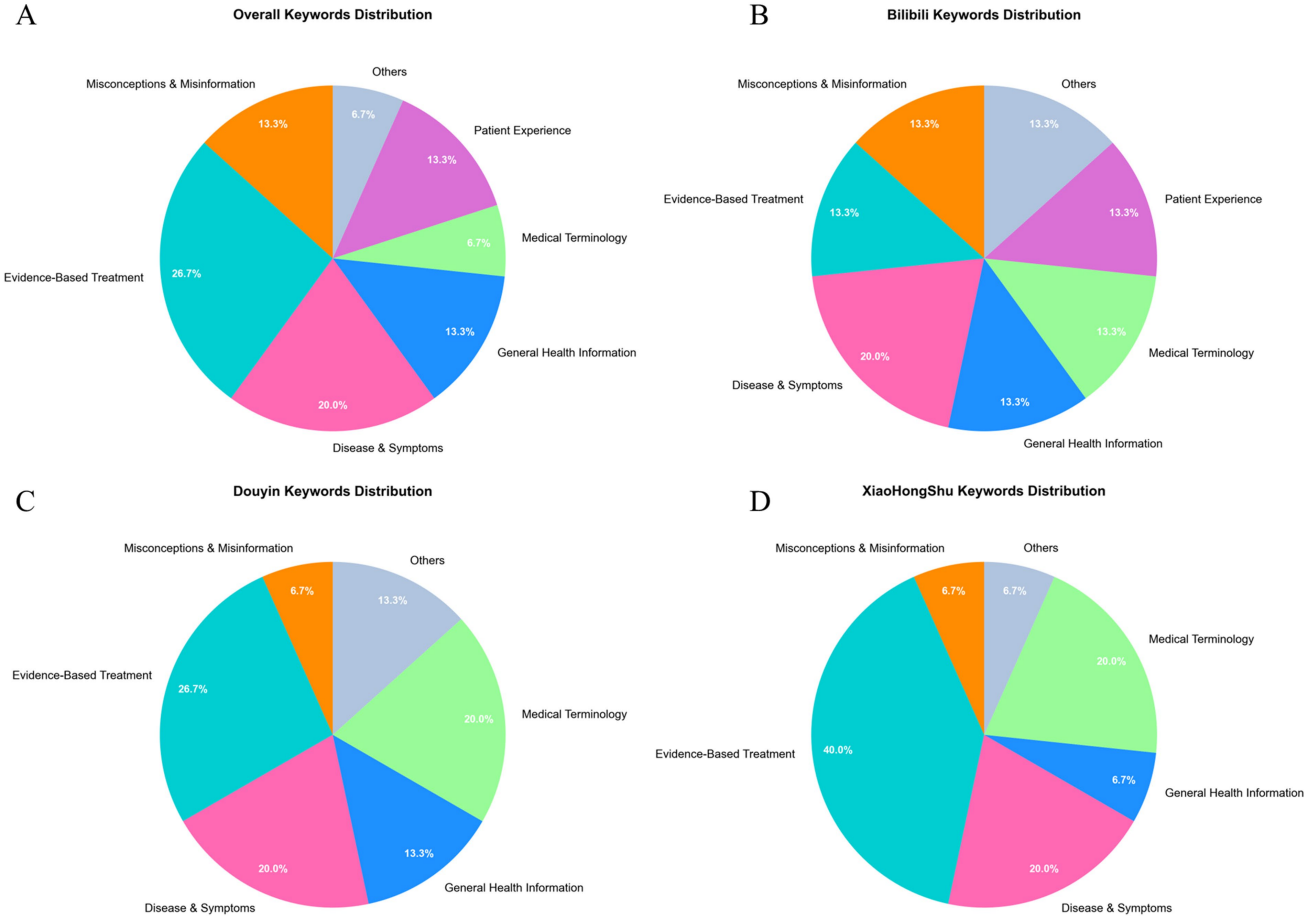
Distribution of keyword categories related to denosumab plus PD-1/PD-L1 therapy across short-video platforms. Pie charts **(A–D)** depict the relative proportions of seven keyword categories extracted from 402 videos across four datasets: overall (*n* = 402), Bilibili (*n* = 222), Douyin (*n* = 105), and Xiaohongshu (*n* = 75). Keywords were identified using a hybrid approach combining the Doubao AI model (doubao-seed-1.6; context length 256 k tokens; temperature 0.3; max output 4,096 tokens), natural language processing techniques, and rule-based categorization. Seven keyword categories were defined based on established health information quality frameworks: 1. Evidence-based treatment. 2. Disease and symptoms. 3. General health information. 4. Medical terminology. 5. Patient experience. 6. Misconceptions and misinformation. 7. Others. Percentages represent the relative frequency of each category within each platform dataset. Data processing was conducted in Python (Pandas 1.3.5, NumPy 1.21.6), and visualizations were generated using Matplotlib 3.5.3. The distribution patterns show that evidence-based treatment content accounts for approximately 20.0–26.7% of keywords, whereas misconceptions and misinformation range from 6.7 to 13.3%, highlighting platform-level differences in scientific rigor and informational accuracy.

Keyword-based thematic analysis further indicated a lack of professional depth. Evidence-based treatment information constituted only 20.0–26.7% of content. Disease symptom descriptions accounted for 20.0% general health information for 13.3% and patient experiences for 13.3%. Although medical terminology appeared in 6.7–20.0% of videos most lacked accurate explanations of underlying mechanisms. Misinterpretations and misinformation were observed in 6.7–13.3% of content varying among platforms (Figure 11).

**Figure 12:**
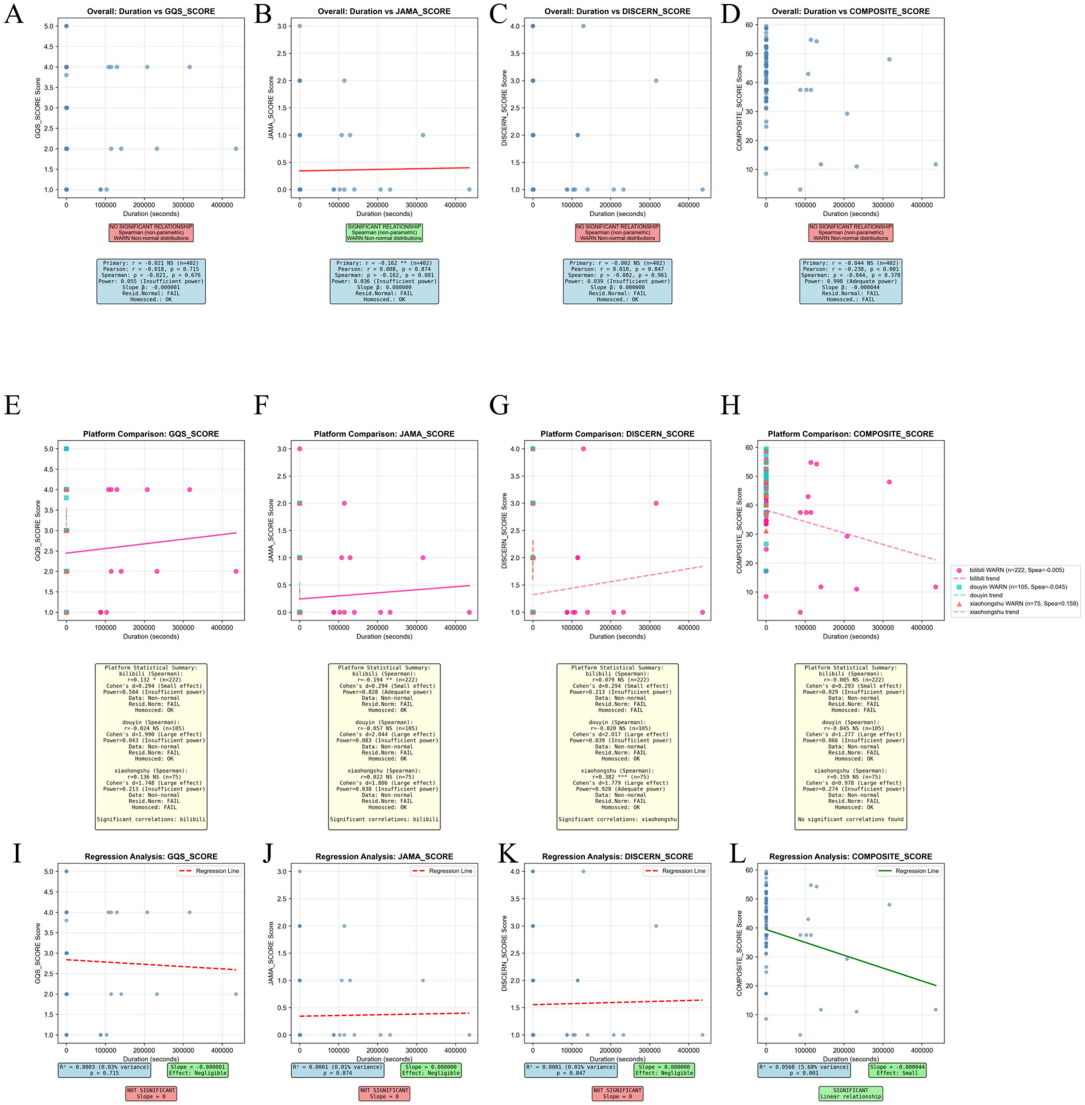
Multi-platform correlation analysis between video duration and quality scores. First row (overall correlations). **(A)** Duration vs. GQS. Spearman analysis shows no significant correlation (r = −0.021, *p* = 0.676; *n* = 402), indicating that longer videos are not associated with higher global quality. **(B)** Duration vs. JAMA score. A weak but statistically significant negative correlation is observed (Spearman r = −0.162, *p* = 0.001), suggesting that longer videos may be modestly associated with lower reliability by JAMA criteria. **(C)** Duration vs. DISCERN. No significant association (Spearman r = −0.002, *p* = 0.961), implying that video length does not meaningfully influence DISCERN scores. **(D)** Duration vs. composite score. Although Pearson analysis indicates a weak negative relationship (r = −0.238, *p* < 0.001), Spearman correlation is non-significant (r = −0.044, *p* = 0.378), suggesting only a small effect of duration on the composite index. Second row (platform-stratified analyses): **(E–H)** Platform-specific Spearman correlations between video duration and quality indicators (GQS, JAMA, DISCERN, composite score) for Bilibili (*n* = 222), Douyin (*n* = 105), and Xiaohongshu (*n* = 75). Significant associations are limited and platform specific: Bilibili shows a weak positive correlation between duration and GQS, and a weak negative correlation with JAMA; Xiaohongshu exhibits a moderate positive correlation between duration and DISCERN; No robust or consistent correlation patterns are observed on Douyin. Third row (regression visualizations): **(I–L)** Linear regression plots illustrate the relationships between duration and GQS, JAMA, DISCERN, and composite scores, respectively. R^2^ values for GQS, JAMA, and DISCERN are near zero, indicating negligible explanatory power of duration for these metrics. For the composite score, R^2^ = 0.0568 (*p* < 0.001), indicating a statistically significant but small effect, with longer videos associated with slightly lower composite quality scores. Overall, these results suggest that video duration is not a reliable predictor of content quality across platforms.

Analysis of video duration demonstrated no significant association with quality. Duration showed no correlation with GQS (r = −0.021, *p* = 0.676) or DISCERN (r = −0.002, *p* = 0.961), and a slight negative correlation with JAMA scores (r = −0.162, *p* = 0.001) (Figures 12A–D). Platform-stratified analyses suggested only isolated associations: Bilibili showed a weak positive correlation with GQS (r = 0.132, *p* < 0.05), while Xiaohongshu demonstrated a moderate correlation with DISCERN (r = 0.382, *p* < 0.001) (Figures 12E–H).

The composite score and duration showed a weak negative association (r = –0.238, *p* < 0.001, R^2^ = 0.0568), indicating that longer content does not necessarily translate into higher-quality information (Figures 12I–L).

### 3.5 User engagement patterns and their relationship with content quality

User engagement was largely decoupled from content quality. Quality scores showed only negligible correlations with log-transformed “likes” counts (Pearson r = 0.062; Spearman *ρ* = 0.047; R^2^ = 0.004), indicating that videos with high engagement were not necessarily high-quality medical resources (Figure 5D). Similar patterns were observed across other engagement metrics: comments (r = 0.043), shares (r = 0.051), and favorites (r = 0.039) all showed non-significant associations with content quality (Figure 5C).

There were statistically significant variations in engagement across platforms. Douyin demonstrated the highest participation levels: median likes = 207 (range: 5–5,619), comments = 18 (range: 0–738), shares = 57 (range: 0–3,275), and favorites = 92 (range: 0–5,870). Despite better performance than other platforms, Douyin content remained far below professional standards (GQS 3.40 ± 0.85; JAMA 0.50 ± 0.62). Bilibili and Xiaohongshu had significantly lower engagement (all *p* < 0.001 vs. Douyin) (Table 1; Figures 6E–H).

Distribution analysis of engagement indicators demonstrated platform-specific interaction patterns: Bilibili exhibited a long-tail distribution, Douyin showed high centralization, while Xiaohongshu displayed the greatest uniformity (Figure 5C).

### 3.6 Inter-platform heterogeneity and reliability validation

**Figure 13:**
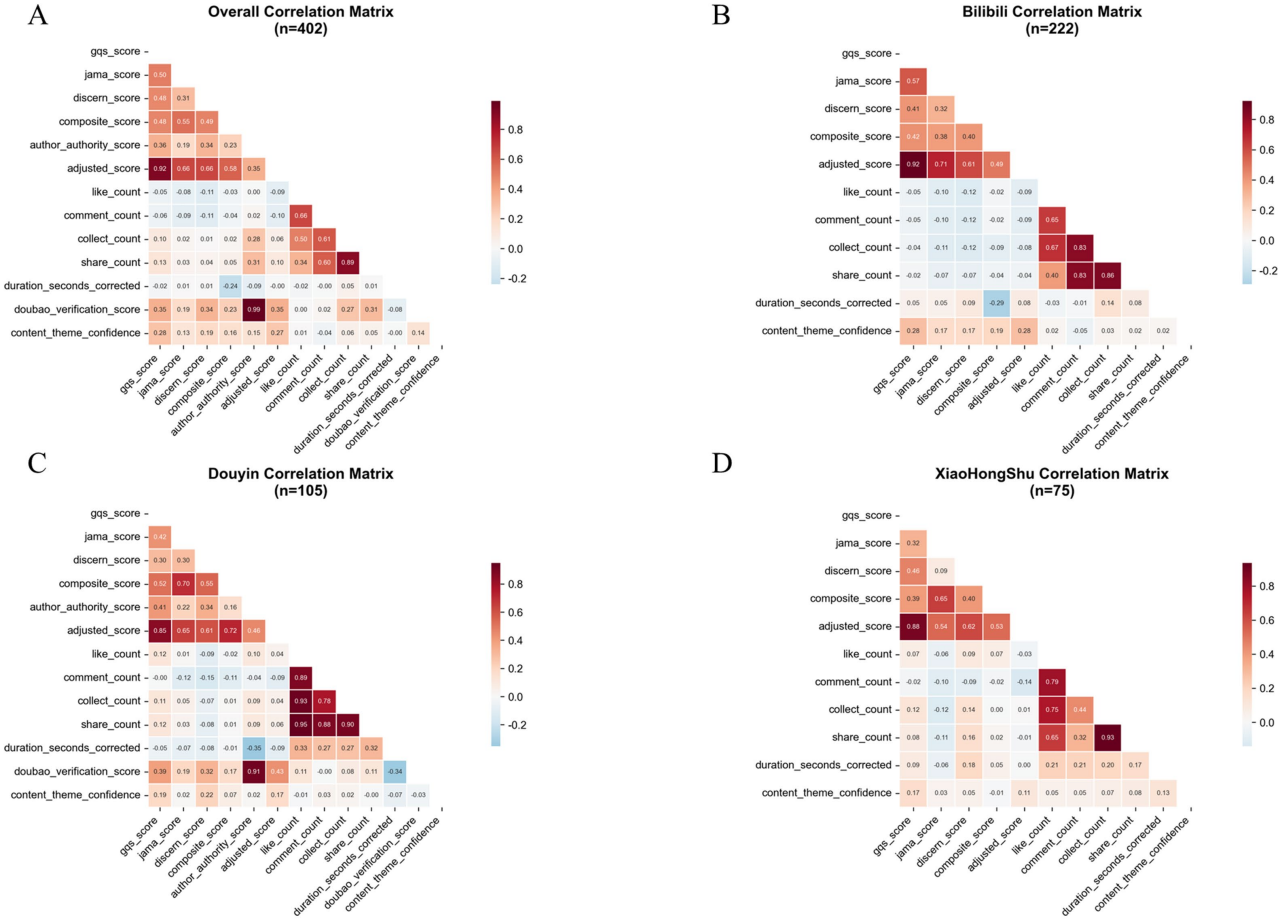
Correlation heatmaps of quality, engagement, and verification indicators across short-video platforms. Four Pearson correlation matrices summarize relationships among 14 key variables in: **(A)** overall dataset (*n* = 402), **(B)** Bilibili (*n* = 222), **(C)** Douyin (*n* = 105), and **(D)** Xiaohongshu (*n* = 75). Variables include quality indicators (GQS, JAMA, DISCERN, composite score, adjusted score), engagement metrics (likes, comments, favorites, shares), duration (duration_seconds_corrected), and platform-specific verification indicators (Doubao verification score, author authority score, content theme confidence). Color intensity ranges from deep blue (strong negative correlation, r = −1.0) to deep red (strong positive correlation, r = 1.0), with white representing no correlation (r = 0). Key findings: 1. Overall dataset: strongest correlations are observed between author authority score and Doubao verification score (r = 0.990, *p* < 0.0001), and between GQS and adjusted score (r = 0.917, *p* < 0.0001). 2. Platform-specific availability: Doubao verification and authority scores are non-applicable (zero-inflated) for Bilibili and Xiaohongshu, indicating that AI-based verification is a unique feature of Douyin. 3. Bilibili: strongest internal consistency among quality metrics (GQS vs. adjusted score, r = 0.922) and high correlation among engagement metrics (collect_count vs. share_count, r = 0.860). 4. Douyin: distinct verification structure with strong coupling between author authority and verification scores (r = 0.911), and the highest correlation among engagement metrics (like_count vs. share_count, r = 0.949). 5. zXiaohongshu: pronounced clustering of engagement indicators (collect_count vs. share_count, r = 0.934) and strong correlation between GQS and adjusted score (r = 0.881). Statistical significance is denoted by *p < 0.05, **p < 0.01, ***p < 0.001. All correlations were estimated using two-sided Pearson tests (SciPy ≥1.7.0), with heatmaps generated in Matplotlib 3.5.3 using a “coolwarm” color scheme. These patterns highlight substantial inter-platform differences in how content quality, creator authority, engagement, and AI verification are structurally linked.

Platform-specific analyses indicated fundamental differences in quality control mechanisms. The correlation matrix revealed distinct variable association structures across platforms (Figure 13). The most striking contrast was in verification scores: only Douyin implemented a systematic verification system (mean 78.581±25.315; median 90.000), whereas Bilibili and Xiaohongshu uniformly displayed zero verification, suggesting an absence of structured content validation (Figures 6K, 9H).

Multi-dimensional reliability analysis showed varying degrees of consistency across platforms. JAMA scores demonstrated moderate correlations with GQS (r = 0.504, *p* < 0.001), DISCERN (r = 0.308, *p* < 0.001), and composite scores (r = 0.547, *p* < 0.001) (Figures 9D–F). Among credibility indicators, speech recognition confidence was highest (0.940 ± 0.068), whereas author authority confidence (0.639 ± 0.273) and topic classification confidence (0.682 ± 0.162) were comparatively lower, reflecting reliability variations across AI-assisted assessments (Figures 9C, I).

Distinct correlation patterns further highlighted platform-level system differences: Douyin: exceptionally high association between creator authority and verification score (r = 0.911); Bilibili: strongest internal consistency among quality indicators (GQS vs. adjusted score: r = 0.922); Xiaohongshu: closest clustering of engagement metrics (collect_count vs. share_count: r = 0.934) (Figure 13). Collectively, these divergences reflect structural disparities in platform ecosystems, user demographics, and algorithmic dissemination mechanisms, which exert a systematic influence on the quality of health information distribution.

## 4 Discussion

### 4.1 Summary of key findings

This study conducted a systematic quality assessment of 402 videos concerning denosumab combined with PD-1/PD-L1 immunotherapy for lung cancer bone metastasis across three leading Chinese short-video platforms. The overall findings revealed a markedly low quality of information, substantially below accepted medical communication standards (54) and professional digital health information criteria (55).

All three core quality indicators demonstrated significant deficiencies: the mean GQS score was only 2.84 ± 1.06 (out of 5; pass rate 56.8%), the mean JAMA score was 0.34 ± 0.57 (out of 4; pass rate 8.5%), and the revised DISCERN score averaged 1.55 ± 0.69 (out of 5; pass rate 31.0%). These results indicate systematic deficiencies in immunology-related information dissemination, raising concerns regarding the risk of patients being exposed to incomplete or misleading knowledge.

Quality differences between platforms exhibited small-to-moderate effect sizes (Cohen’s d = 0.6–0.8). Douyin performed relatively better (GQS: 3.40 ± 0.85; JAMA: 0.50 ± 0.62; DISCERN: 1.90 ± 0.67), whereas Bilibili performed the worst (GQS: 2.45 ± 1.03; JAMA: 0.25 ± 0.51; DISCERN: 1.33 ± 0.61). These disparities may be attributable to heterogeneity in platform demographics, content moderation policies, and algorithmic recommendation designs (55, 56), which directly affect user exposure to credible health information and may further reinforce pre-existing biases.

Moreover, variations in machine-learning-based recommendation systems across platforms likely exacerbate unequal information distribution (57), creating potential hindrances to informed clinical communication, treatment decision-making, therapeutic adherence, and adverse event reporting.

### 4.2 Platform-driven cognitive biases and clinical safety risks associated with immunotherapy

Short-video platforms have rapidly become primary health information sources for the general public and patients, influencing health perceptions and—indirectly—behavior. According to dual-process cognitive models, individuals exposed to vast online information tend to rely either on: (1) heuristic cues (e.g., likes, creator identity), or (2) systematic processing with higher engagement (58, 59).

However, within short-video ecosystems, heuristic-driven judgment dominates: users often rely on popularity signals rather than informational accuracy (58, 60). Individuals with lower health literacy are particularly vulnerable to persuasive but oversimplified narratives (61–63), while repetitive short-form viewing diminishes cognitive readiness (64), promotes reactive attention (64), fragments memory (65), and increases misunderstanding.

Algorithm-driven reinforcement of familiar content further leads to “algorithmic echo chambers,” amplifying pre-existing beliefs while narrowing exposure to diverse viewpoints (66, 67). Such reinforcement has measurable effects on self-management behaviors (68). Critically, most platform algorithms prioritize engagement over quality (69–71), intensifying confirmation bias and shaping risky health decision-making (72).

Our findings align with these theoretical mechanisms: user engagement was almost entirely decoupled from content quality (R^2^ = 0.004; r = 0.062), demonstrating that recommendation systems systematically elevate popular but potentially inaccurate content. As a result, misconceptions regarding denosumab or PD-1/PD-L1 inhibitors may be algorithmically amplified, creating information inequity (56) and cognitively influencing clinical expectations and decisions (57).

Low-quality videos commonly presented overly simplified mechanisms such as: *“Denosumab inhibits bone destruction and protects bone,”* without explaining the TRAF6–NF-κB/MAPK–NFATc1 signaling cascade downstream of RANKL-RANK interaction (73, 74). PD-1/PD-L1 pathway explanations lacking mechanistic clarity on ITIM/ITSM-mediated SHP2 recruitment and suppression of TCR/CD28 signaling (12).

Platform disparities may further reinforce such biases: Douyin vs. Bilibili scores (GQS: 3.40 ± 0.85 vs. 2.45 ± 1.03; JAMA: 0.50 ± 0.62 vs. 0.25 ± 0.51; all *p* < 0.001; Cohen’s d = 0.6–0.8). Even the highest JAMA score (12.5% of full score) remains far below basic medical communication standards (54, 55).

Additionally, most content was produced by patients (52.2%), while only 25.6% originated from medical professionals. When personal anecdotes are algorithmically generalized, subjective or inaccurate beliefs may gain disproportionate influence, increasing the potential for harmful treatment interpretations and safety risks.

Building upon cognitive-behavioral theory, the disparities observed across platform algorithms and content quality may reinforce or exacerbate cognitive biases among short-video users, particularly in relation to mechanisms of denosumab–immunotherapy combinations (75). Repetitive exposure to content emphasizing selective aspects of immunology may foster persistent cognitive errors—such as availability bias and misattribution—which are known contributors to diagnostic inaccuracy (28). This phenomenon is consistent with the algorithmic echo chamber, whereby users’ pre-existing beliefs are continuously reinforced (66, 67), negatively impacting their understanding of treatment complexity (76).

Clinically relevant safety concerns may arise from such distorted perceptions. For instance, insufficient attention to calcium/vitamin D supplementation and serum calcium monitoring could elevate the risk of hypocalcemia during denosumab therapy, which has been reported in 15–30% of patients due to inadequate supplementation, with severe cases resulting in tetany and arrhythmias (77). Similarly, inadequate awareness of the necessity for dental assessment may increase the risk of medication-related osteonecrosis of the jaw (MRONJ). Evidence shows the incidence of MRONJ can reach 1.8–12.6% in patients without adequate oral screening and preventive care (78–81), and is further heightened in those with pre-existing oral infections or recent tooth extractions (82). However, comprehensive evaluation and prophylaxis can reduce incidence to 0.8–4.4% (79, 83). Neglecting pre-treatment dental evaluation therefore jeopardizes accurate assessment of the risk–benefit ratio of combined immunotherapy and can negatively influence clinical prognosis (84).

Moreover, combining denosumab with PD-1/PD-L1 inhibitors increases the likelihood and severity of immune-related adverse events (irAEs), with endocrine irAEs showing a 15–20% increased risk (85). Over-simplified messaging emphasizing “bone protection” may lead to inadequate endocrine monitoring and delayed irAE recognition, further elevating complication risks (86). Misattribution can also occur when patients incorrectly link symptoms such as bone or dental pain solely to treatment toxicity. Such associative misattribution not only interferes with appropriate clinical decision-making but may lead to inflated toxicity signals and substantial heterogeneity in safety reporting in clinical trials (87).

### 4.3 Strengths and limitations

Although AI-assisted scoring improved evaluation efficiency, limitations persisted in semantic interpretation of complex immunological concepts and contextual reasoning. The inherently individualized nature of immunotherapy—including tumor heterogeneity, immune microenvironment variation, and host immune status—adds challenges to standardized assessment (88). The cross-sectional design prevented assessment of dynamic changes in patient cognition or cumulative information exposure, and real-world impacts on clinical decision-making or treatment outcomes were not directly evaluated, necessitating future longitudinal validation (89).

Furthermore, this study focused solely on Chinese-language platforms, limiting generalizability to diverse cultural contexts where perceptions of immunotherapy may differ (90). While 10% blind quality auditing met ISO recommendations, potential sampling bias cannot be completely excluded. Additionally, the three-tier keyword strategy—despite its systematic design—may have overlooked high-quality videos with more colloquial terminology, potentially under-estimating platform performance.

Despite these limitations, this study represents the first systematic evaluation of denosumab-immunotherapy information quality from an immunology perspective on major Chinese short-video platforms. The findings illuminate critical issues of accuracy deficits, limited professional involvement, and cognitive risk in digital health communication. These results highlight the urgent need to establish professional immunology-based standards and regulatory frameworks for digital health information to safeguard patients’ right to reliable guidance.

From a clinical translation perspective, this study provides actionable implications for immuno-oncology practice: (1) clinicians should proactively contribute to digital science communication to correct misinformation, (2) digital exposure should be systematically assessed during consultations, and (3) healthcare systems should develop authoritative hospital-led patient-education platforms.

Collectively, these strategies may enhance digital health literacy, strengthen shared decision-making, and ultimately improve patient-centered immunotherapy outcomes.

### 4.4 Future directions

Future studies should adopt prospective cohort designs to track digital health information exposure throughout diagnosis and treatment, evaluating its effects on immunotherapy decision-making, adherence, and clinical outcomes (91). Interventional studies grounded in immunology-related cognitive theory should compare educational strategies—e.g., visualization, case-based learning, interactive Q&A—to enhance patients’ immunological understanding (92), combined with AI-driven personalized education recommendation systems.

Importantly, targeted interventions must correct misconceptions about denosumab and immune checkpoint inhibitors, while establishing a standardized patient-education pathway that ensures appropriate supplementation, toxicity monitoring, and timely reporting of adverse events. Development of unified cognition-assessment frameworks and structured education protocols will be essential to strengthen patient capacity for irAE recognition and management (93).

## 5 Conclusion

This study provides the first evidence demonstrating a substantial deficiency in the quality of information regarding denosumab combined with immunotherapy for lung cancer bone metastasis on major Chinese short-video platforms. A pronounced disconnect was observed between content quality and user engagement, suggesting that algorithmic recommendation systems may systematically amplify inaccurate or oversimplified immunological information. Such distortions may lead to clinically relevant cognitive risks, including inadequate monitoring for hypocalcemia, insufficient MRONJ prevention, and misinterpretation of irAE-related symptoms, while also introducing bias into adverse-event reporting and clinical research outcomes.

These findings underscore the urgency of establishing standardized regulatory frameworks and professional oversight mechanisms to ensure the accuracy and safety of digital health communication, thereby improving treatment literacy, reducing misinformation-driven harm, and ultimately enhancing patient-centered immunotherapy outcomes.

## Data Availability

The raw data supporting the conclusions of this article will be made available by the authors, without undue reservation.
