# Supplementary material for: The theoretical impact of AI-based quality evaluation of short-video health information on public cognition and treatment adherence: a case study of denosumab combined with PD-1/PD-L1 therapy for lung cancer bone metastasis
Source: Front Public Health. 2026 Jan 6;13:1724546. doi: 10.3389/fpubh.2025.1724546 (PMC12816342; doi:10.3389/fpubh.2025.1724546)
Supplement: Supplementary file 1 [file Data_Sheet_1.docx]

Supplementary Material

# Parameter Configuration Details

1. Study Design Parameters

1.1 Study Type and Design

Study Design: Cross-sectional observational study

Reporting Guideline: STROBE Statement

Research Topic: Impact of denosumab combined with PD-1/PD-L1 inhibitors on bone metastases in lung cancer

Search Keywords: "Impact of denosumab combined with PD-1/PD-L1 inhibitors on bone metastases in lung cancer"

1.2 Sample Size and Effect Size

Initial Dataset: 1,369 videos

Final Sample: 402 videos

Platform Distribution:

Bilibili: 222 videos (55.2%)

Douyin: 105 videos (26.1%)

Xiaohongshu: 75 videos (18.7%)

Planned effect size for power analysis: Cohen’s f² = 0.15 (medium; equivalent to R² ≈ 0.13).

Statistical Power: 1-β = 0.80

Significance Level: α = 0.05

Test Type: Two-tailed

1.3 Platform Selection Criteria

Bilibili: >270 million monthly active users

Xiaohongshu: >200 million monthly active users

Douyin: >600 million daily active users

Retrieval Scope: All historical data on each platform; no time restriction

2. Ethical Considerations

2.1 Ethics and Compliance

Study Type: Public social media content analysis

Human Subjects: Not involved

Clinical Data: Not involved

Animal Use: Not involved

Ethics Approval: Not required

Anonymization: Fully anonymized data processing

Personal Identifiers: No collection or usage of personally identifiable information

3. Data Collection Parameters

3.1 Relevance Scoring Framework

Evaluation Method: Three-tiered keyword relevance scoring

Tier 1 (High Weight): denosumab, bone metastases, lung cancer with bone metastases

Tier 2 (Medium Weight): immunotherapy, PD-1, PD-L1, checkpoint inhibitors, lung cancer, combination therapy

Tier 3 (Low Weight): bone lesions, osteoporosis, targeted therapy, clinical trials, evidence-based medicine

Inclusion Threshold: Relevance score ≥ 1

3.2 Relevance Scoring Criteria

3 points: Contains both Tier 1 and Tier 2 keywords

2 points: Contains Tier 1 only

1 point: Contains only Tier 2 or Tier 3

0 points: No relevant keywords

3.3 Inclusion and Exclusion Criteria

Inclusion:

Content related to denosumab or lung cancer bone metastasis treatment

Video duration ≥15 seconds

Chinese language, clear audio

Human-rated relevance score ≥1

Complete transcript and platform metadata

Exclusion:

Pure commercial/advertisement content

Duplicates or reposted content

Inaudible or unclear audio

Evident misinformation

Incomplete transcription or metadata

4. Video Classification Parameters

4.1 Uploader Categories

Domain Experts: Oncology, orthopedics, or related specialists

Other Medical Professionals: General physicians, pharmacists, nurses

Patients and Families: Individuals with disease experience

Others: Science communicators, media accounts, non-medical users

4.2 Identity Verification

Certification System: Automated identification system

Verification Method: AI-based name and content analysis

API: Doubao API for professional background validation

Confidence Score: 0–1 scale

Douyin-Specific Module: Customized program to fix and enhance metadata

5. Quality Assessment Parameters

5.1 AI Evaluation System Configuration

LLM Model: doubao-seed-1.6

Architecture: Modular framework

Core Modules: Evaluation engine + integrated chart generator

Context Length: 256k tokens

Scoring Standards: GQS, JAMA benchmark, (m)DISCERN (revised DISCERN)

5.2 GQS (Global Quality Scale)

Scale: 1–5 points

Dimensions: Content accuracy, completeness, clarity, structure, practical value

Reference: Bernard et al., 2007

5.3 JAMA Information Benchmark

Scale: 0–4 points

Criteria: Authorship, attribution, sources, currency

Reference: Silberg et al., 1997

5.4 Revised DISCERN (mDISCERN)

Scale: 1–5 points

Dimensions: Treatment options, risk–benefit analysis, info quality, decision support, overall assessment

Reference: Charnock et al., 1999

5.5 Implementation Details

AI Scoring: Each video evaluated independently 3 times

Re-Evaluation Trigger: If score variance >1

Human Review: Performed by domain experts

Quality Control: Outlier detection + manual verification of 10% sample

5.6 Composite Quality Score Formula

Formula: Overall Score = 0.4 × GQS_normalized + 0.3 × JAMA_normalized + 0.3 × DISCERN_normalized

Weighting Justification:

GQS (Accuracy/Clarity): 40%

JAMA (Reliability): 30%

DISCERN (Treatment Info): 30%

5.7 Technical Components

Speech-to-Text Engine: Custom processor with medical terminology correction

Vocabulary Mapping: Drugs, diagnoses, treatment concepts

Identity Recognizer: Detects institutional affiliation and medical credentials

Data Security: Anonymization, access control, activity logging

6. Statistical Analysis Parameters

6.1 Technical Environment

Language: Python ≥3.9

Core Libraries:

pandas, numpy, scipy, matplotlib, seaborn, scikit-learn

6.2 Visualization Settings

Generator: Integrated academic chart builder

Processing: Multithreaded

Output Standard: DPI = 300 (publication-grade)

Color Scheme: Color-blind-friendly palette

6.3 Descriptive Statistics

Central Tendency: Mean, median, mode

Dispersion: SD, IQR, CV

Distribution: Skewness, kurtosis, Shapiro–Wilk test

Stratified Analysis: By platform (Bilibili, Douyin, Xiaohongshu)

Keyword Analysis: NLP-based topic modeling

6.4 Inferential Statistics

Significance Level: α = 0.05

Platform Comparisons:

Multi-group: Kruskal–Wallis test

Pairwise: Mann–Whitney U test

Categorical: Chi-square test

Test Type: Two-tailed

Threshold for Significance: P < 0.05

6.5 Correlation Analysis

Normal Data: Pearson coefficient

Non-normal Data: Spearman correlation

Strength Interpretation:

Weak: |r| < 0.3

Moderate: 0.3 ≤ |r| < 0.7

Strong: |r| ≥ 0.7

P-Adjustment: FDR correction

6.6 Effect Size Evaluation

Metric: R² coefficient of determination

Effect Size Interpretation:

Small: R² < 0.13

Medium: 0.13 ≤ R² < 0.26

Large: R² ≥ 0.26

6.7 Regression Analysis

Model: Multiple linear regression

Diagnostics: Residual analysis

Statistical Outputs: Automated report generation

Visualization: Baseline tables, heatmaps, histograms

7. Doubao API Configuration (LLM)

7.1 Base API Settings

Endpoint: https://ark.cnbeijing.volces.com/api/v3/chat/completions

Model: doubao-seed-1.6

Max Tokens: 256k

Timeout: 30 seconds

Retry Logic: Max 3 retries; 2s delay

Rate Limit: 60 requests/min

7.2 Model Parameters

Temperature: 0.3

Max Tokens (per response): 4096

Top-p: 0.9

Frequency Penalty: 0.0

Presence Penalty: 0.0

8. Data Processing and Quality Control

8.1 Data Integrity Assurance

Transcription: Manual correction to supplement ASR

Data Imputation: Addressed missing data (except Xiaohongshu view counts)

Data Cleaning: Excluded videos lacking complete transcripts

8.2 Platform-Specific Fixes

Douyin Certification: Custom-built module for metadata correction

Physician Verification: Based on Douyin’s certification system

Consistency Check: Cross-platform metadata harmonization

8.3 Quality Control Measures

Outlier Detection: Implemented and corrected

Random Validation: 10% of data manually reviewed

Dual Verification: AI evaluation + expert audit

Data Security: Compliance via anonymization, access control, and audit logging

**Prompts：**

1. GQS Scoring Prompt

Please act as a medical content quality expert and evaluate the quality of the following medical video based on the GQS (Global Quality Score) criteria.

Video information:

Title: {title}

Author: {author_name}

Platform: {platform}

Publication date: {publish_time}

Number of likes: {like_count}

Number of comments: {comment_count}

Please provide a final score along with a brief justification to support expert review and subsequent refinement.

1. JAMA Scoring Prompt

Please act as an expert in medical information credibility and assess the transparency and reliability of the following medical video based on the JAMA Benchmark Criteria.

Video information:

Title: {title}

Author: {author_name}

Platform: {platform}

Publication date: {publish_time}

Please provide a final score and a concise explanation to support expert review and further adjustments.

1. DISCERN Scoring Prompt

Please act as an evaluator specializing in treatment information quality and assess the following medical video using the DISCERN criteria.

Video information:

Title: {title}

Content summary (≤ 500 words): {video_content}

Author: {author_name}

Please provide a rating along with a brief rationale to facilitate expert validation and subsequent modification.

1. Variable Placeholder Definition

{title}: Video title

{author_name}: Name of the uploader or content creator

{platform}: Platform where the video was posted

{publish_time}: Video upload date

{like_count}: Number of likes

{comment_count}: Number of comments

{video_content}: Main text or summarized description of the video (required for DISCERN evaluation)
